# Characterizing the photodegradation-induced release of volatile organic compounds from bottled water containers

**DOI:** 10.1016/j.eehl.2024.01.005

**Published:** 2024-02-08

**Authors:** Ruijuan Liu, Zhianqi Liao, Jing Zheng, Xinni Wu, Zongyi Tan, Huase Ou

**Affiliations:** a Guangdong Key Laboratory of Environmental Pollution and Health, School of Environment, Jinan University, Guangzhou 511443, China; b State Environmental Protection Key Laboratory of Environmental Pollution Health Risk Assessment, South China Institute of Environmental Sciences, Ministry of Ecology and Environment, Guangzhou 510655, China; c Key Laboratory of Philosophy and Social Science in Guangdong Province of Community of Life for Man and Nature, Jinan University, Guangzhou 511443, China

**Keywords:** Plastic contaminants, Volatile organic compounds, Drinking water safety, Sunlight, Ultraviolet

## Abstract

While plastic water bottles are known to potentially release various volatile organic compounds (VOCs) when exposed to light, existing knowledge in this field remains limited. In this study, we systematically examined the composition, yield, and toxicity of VOCs released from six plastic containers obtained from different continents under UV-A and solar irradiation. After light exposure, all containers released VOCs, including alkanes, alkenes, alcohols, aldehydes, carboxylic acids, aromatics, etc. The 1#, 3#, 4#, 5#, and 6# containers exhibited 35, 32, 19, 24 and 37 species of VOCs, respectively. Specifically, the 2# container released 28 and 32 series of VOCs after 1-day (short-term) and 7-day (long-term) UV-A irradiation, respectively, compared to 30 and 32 species under solar irradiation. Over half of the VOCs identified were oxidized compounds alongside various short-chain hydrocarbons. Significant differences in VOC compositions among the containers were observed, potentially originating from light-induced aging and degradation of the polyethylene terephthalate structure in the containers. Toxicological predictions unveiled distinctive toxic characteristics of VOCs from each container. For example, among the various VOCs produced by the 2# container, straight-chain alkanes like n-hexadecane (544-76-3) were identified as the most toxic compounds. After long-term irradiation, the yield of these toxic VOCs from the 2# container ranged from 0.11 ng/g to 0.79 ng/g. Considering the small mass of a single bottle, the volatilization of VOCs from an individual container would be insignificant. Even after prolonged exposure to light, the potential health risks associated with inhaling VOCs when opening and drinking bottled water appear manageable.

## 1 Introduction

Bottled water, available in different types such as purified water, natural mineral water, and spring water, packaged in either glass or plastic containers, has gained widespread popularity as a drinking water source. The global bottled water market is experiencing rapid growth, with approximately 600 million households consuming bottled water in 2022. Moreover, the consumption of bottled water is increasing at a rate of 5% annually and is projected to reach a volume of around 515 billion liters by 2027 [1]. Over time, consumers have come to believe that bottled water is a safer alternative with fewer contaminants compared to tap water. This perception is often rooted in the adherence of bottled water production to rigorous standards, rendering it widely regarded as purer than tap water. However, it is crucial to acknowledge that plastic bottled water containers can also be a potential source of contamination. Polyethylene terephthalate (PET) stands as the primary material utilized for these plastic containers. Apart from its polymer structure, various additives are integrated to enhance its performance, which may leach into bottled water during storage and usage [2]. In light of these concerns, researchers have invested substantial efforts in assessing the concentration and transfer of pollutants from plastic containers into bottled water, as well as investigating their potential toxicity [[3], [4], [5]].

Most current research has predominantly centered on the examination of additives found in PET, with phthalates emerging as notable candidates for releasing organic compounds from PET bottles [[6], [7], [8]]. Additionally, other endocrine-disrupting compounds, such as hormones [9], bisphenol A [10], and 17β-estradiol [11], have been identified. Toxicological analyses have been conducted as well, with several studies indicating that the organic matter released from PET bottles during storage did not manifest toxic effects [[12], [13], [14]]. While various pollutants have been detected in bottled water, the majority of their concentrations were found to be below established safety thresholds, and their potential toxic effects on the human body were considered negligible. Consequently, the prevailing consensus is that bottled water in PET plastic containers is generally safe.

However, existing research has predominantly concentrated on organic contaminants dissolved in water samples from bottled water containers, overlooking the issue of pollution from volatile organic compounds (VOCs) in the air phase. To date, the sole investigation into air samples from bottled water was conducted by Leivadara et al. [15] nearly fifteen years ago, utilizing an analysis method and VOC detection device with low sensitivity. Consequently, the presence of VOCs in bottled air remains uncertain. Environmental stress, particularly from light exposure, can lead to the degradation and aging of PET containers. Recent studies have underscored the generation of microplastics from aged plastic bottles due to light irradiation and heat stress [16]. Moreover, the aging process of PET containers may transpire during solar water disinfection [17,18]. Our previous research discovered that the aging of polypropylene, polystyrene, and polyvinyl chloride microplastics resulted in the release of numerous VOCs under ultraviolet (UV) irradiation [19]. Therefore, under conditions of light and heat stress, such as exposure to sunlight during transportation and storage in stores, the chemical structure of PET containers may deteriorate, potentially releasing VOCs. Consider a common scenario as an example: inadvertently storing partially consumed bottled water in a car, subjecting the PET container to prolonged solar irradiation and elevated temperatures, which may result in the release of VOCs into the air within the bottle. Consequently, individuals may inhale the contaminated air, potentially exposing themselves to health risks.

The aim of this study is to investigate the release of VOCs from plastic containers and their risks to human health under ambient light stress, specifically UV-A irradiation and solar irradiation. Six commercial bottled water containers from different continents were selected. A non-target screening was performed to identify the VOCs. We examined the variations in VOC product composition among containers from different sources. To assess the potential toxicity of the detected VOCs, toxicological prediction and prioritization using the Toxicological Priority Index (ToxPi), a multicriteria-based ranking method, was conducted. Furthermore, the yield of the top toxic VOCs was determined, and the generation patterns of VOCs in the presence of different solution matrices were investigated. The outcomes of this study are expected to yield valuable data for the comprehensive risk assessment of VOCs released from bottled water containers under light-induced stress.

## 2 Experimental

### 2.1 Materials

Since the materials of almost all bottled water containers were PET, only six different brands of bottled water were selected as representative samples, which covered a variety of source locations (China domestic, Asia, America, Europe, and Oceania) and source types (spring water, distilled water, artesian water, etc.). Their detailed information is listed in Table S1. The bottled water was poured out, and then their outer labels were removed. The containers, after washing with ultrapure water, became fully transparent (Fig. S1). Brand information was marked, and then the containers were sealed and kept in the dark. The standard of mix n-alkane (C6–C40) was obtained from ANPEL (Shanghai, China). Standards of highly toxic VOCs (identified by a method in Section 2.6) were purchased from Sigma (St. Louis, USA). All other reagents were used as received. Ultrapure water was purchased from Fisher (Waltham, MA).

### 2.2 Pre-experiment of containers

Two experimental groups were performed. The purpose of the first group was to analyze the generation of VOCs from the containers without any solution during the irradiation. The empty containers were dried at 40 °C and then sealed. The results of 3.1, 3.2, 3.3, 3.4 were obtained using containers from the first set. The purpose of the second group was to analyze the VOCs from the containers during the irradiation with different solution matrices. Half the volume of the containers was filled with solution. Three types of solution were used: deionized water, mineral water, and soda water (Table S2). Afterward, the containers were sealed. The results of Section 3.5 were obtained using containers from the second set.

**Fig. 1:**
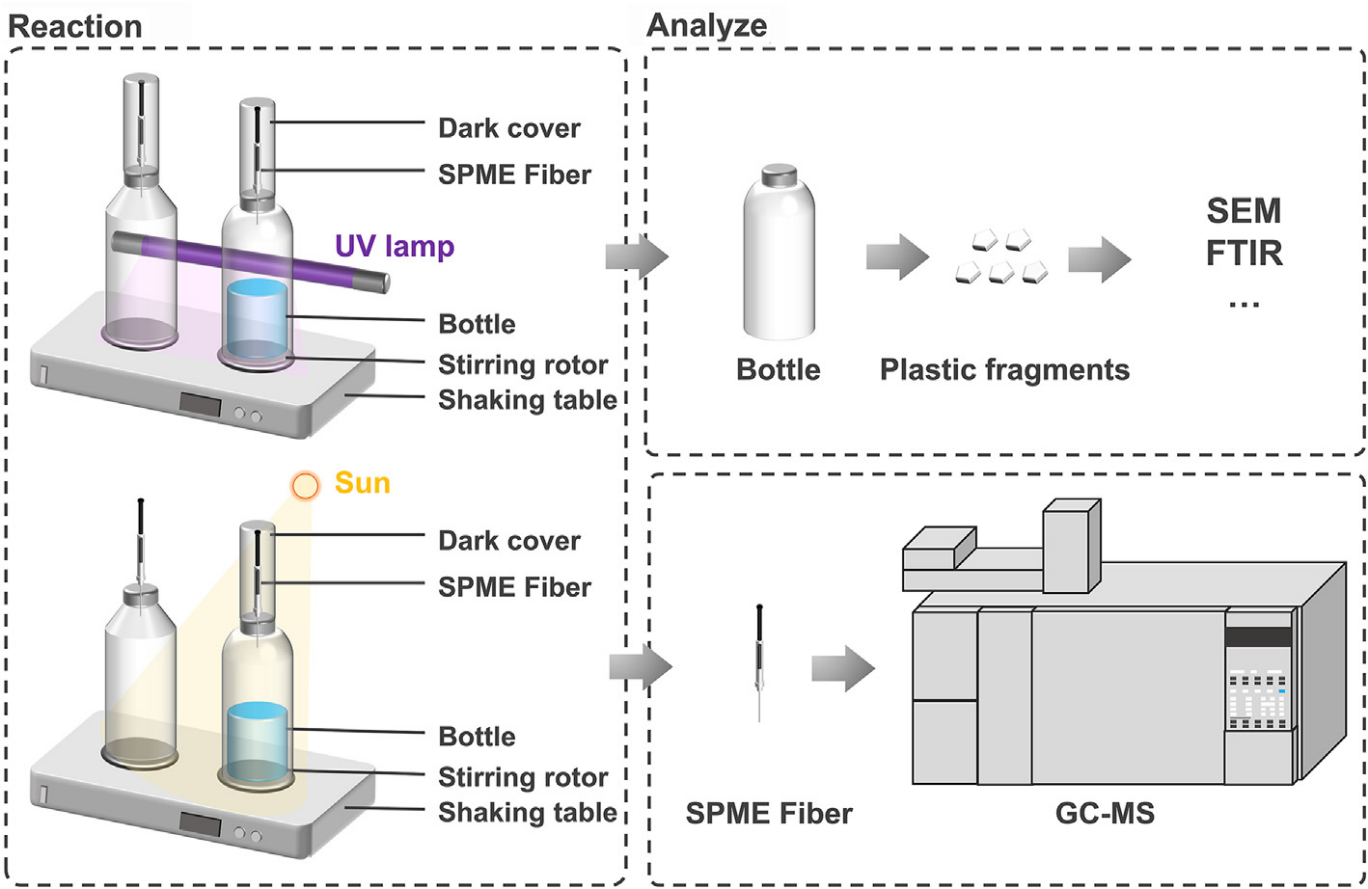
Experimental system diagram. Two groups of pre-treatments were performed. The purpose of the first group was to analyze the generation of VOCs from the containers during the irradiation without any solution. The purpose of the second group was to analyze the VOCs from the containers during the irradiation with different solution matrices. Half the volume of the containers was filled with solution. Two groups of irradiation experiments were conducted under the assumption that the bottled water was exposed to sunlight in a place. The first group was solar treatment, while the second group was UV-A-treated experiment. The VOCs were analyzed by SPME and GC–MS.

Then, a hole with a diameter of 0.2 mm was drilled into the bottle cap and sealed with PTFE parafilm (Fig. 1). The containers were placed on the bench for subsequent irradiation experiments. As a control, empty glass containers (500 mL) were customized using JGS-1 glass (with a transmission ratio of ∼95% at 365 nm), and a 0.2 mm diameter hole was also drilled on the bottle cap with a PTFE parafilm. The control containers were kept on the bench throughout the entire experiment.

### 2.3 UV-A and solar irradiation

Two groups of irradiation experiments were conducted under the assumption that the bottled water was exposed to sunlight in real-world conditions. The first group was solar treatment. The containers were placed directly in the open land and irradiated for 1 day or 7 days. The aim of setting two irradiation times was to compare the difference of VOCs released with ongoing irradiation. The experiment period was carried out in the summer of 2022 over 1 or 7 consecutive sunny days, with the mean temperature at 27 ± 5 °C. The UV-A intensity was measured using a UV radiometer at 8 a.m., 11 a.m., 2 p.m., and 5 p.m. every day (Beijing Normal University Photoelectric Technology, China). An average irradiation intensity at 5.6 W/m^2^ UV-A was measured. The experimental containers were fixed on a shaking table oscillating at 60 rpm, inclined at a 30° angle with respect to the horizontal position, oriented south, and exposed to natural sunlight (normal environmental condition).

The second group was the UV-A-treated experiment, which aimed at identifying the role of UV-A irradiation in VOC generation. Only UV-A radiation was considered since PET does not transmit UV-B radiation [20,21]. It was performed using a customized irradiation module with a low-pressure mercury lamp emitting at 365 nm. Irradiation intensity on the surface of containers was adjusted to 5.6 W/m^2^, which was close to the average UV irradiation (∼5 W/m^2^) in summer in south China [22]. For the long-term samples, an 84-h continuous irradiation was performed over a period of 7 days, with 12 h of irradiation per day. It was 1 day for the short-term experiments. The experimental containers were fixed on a shaking table oscillating at 60 rpm under UV irradiation and room temperature. The irradiation doses used for both light exposure experiments were consistent. For both groups, after experiments, containers were transferred to the dark at room temperature (21–25 °C), and headspace sampling was conducted within 2 h.

In order to avoid any systematic or non-systematic organic contamination caused by experimental instruments and containers, blank container samples without irradiation treatment were added to each group.

### 2.4 Extraction of VOCs

The solid-phase microextraction (SPME) fiber and its outer protective syringe were punctured from the position of the reserved hole into the bottle through the PTFE sealing film. The SPME fiber was extended to adsorb the VOCs in the bottle. The adsorption time was 30 min. After the adsorption, the SPME fiber was retracted into the protective syringe. The entire SPME holder was withdrawn and placed into the gas chromatography (GC) injector port at 250 °C and remained for 180 s for desorption. After each sample injection, fibers were kept inside the SPME needle to prevent possible contamination and were conditioned with helium at 250 °C for 10 min before reuse.

### 2.5 Non-targeted analysis and quantitative analysis

The qualitative and quantitative analyses using mass spectrum and the non-targeted analysis for VOCs were conducted following similar methods in our previous study [19,23].

### 2.6 Toxicity prediction ranking

Since more than 200 VOCs were identified, it was necessary to evaluate their toxicological prioritization. A toxicity prediction ranking using ToxPi software based on the Conditional Toxicity Value (CTV) predictor (https://toxvalue.org/6-CTV/Cover.php) and ToxCast screening library database was conducted following a similar procedure in our previous study [23] (Text S1).

### 2.7 Quality assurance and quality control (QA/QC)

All glassware was thoroughly cleaned with deionized water and ultrapure water in triplicate, followed by heating in Muffle furnaces for 4 h (450 °C) to eliminate any residual organic matter. To remove any organic matter attached to the surface of the containers prior to the irradiation experiment, empty PET containers were immersed in a mixture of ultra-pure water and ethanol for 12 h and then left to dry at ambient room temperature. To ensure the analysis stability of GC–MS, one standard sample was analyzed after every nine experimental samples. Each experimental treatment was conducted in triplicate.

## 3 Results and discussion

### 3.1 Total ion chromatograms of VOCs from containers

**Fig. 2:**
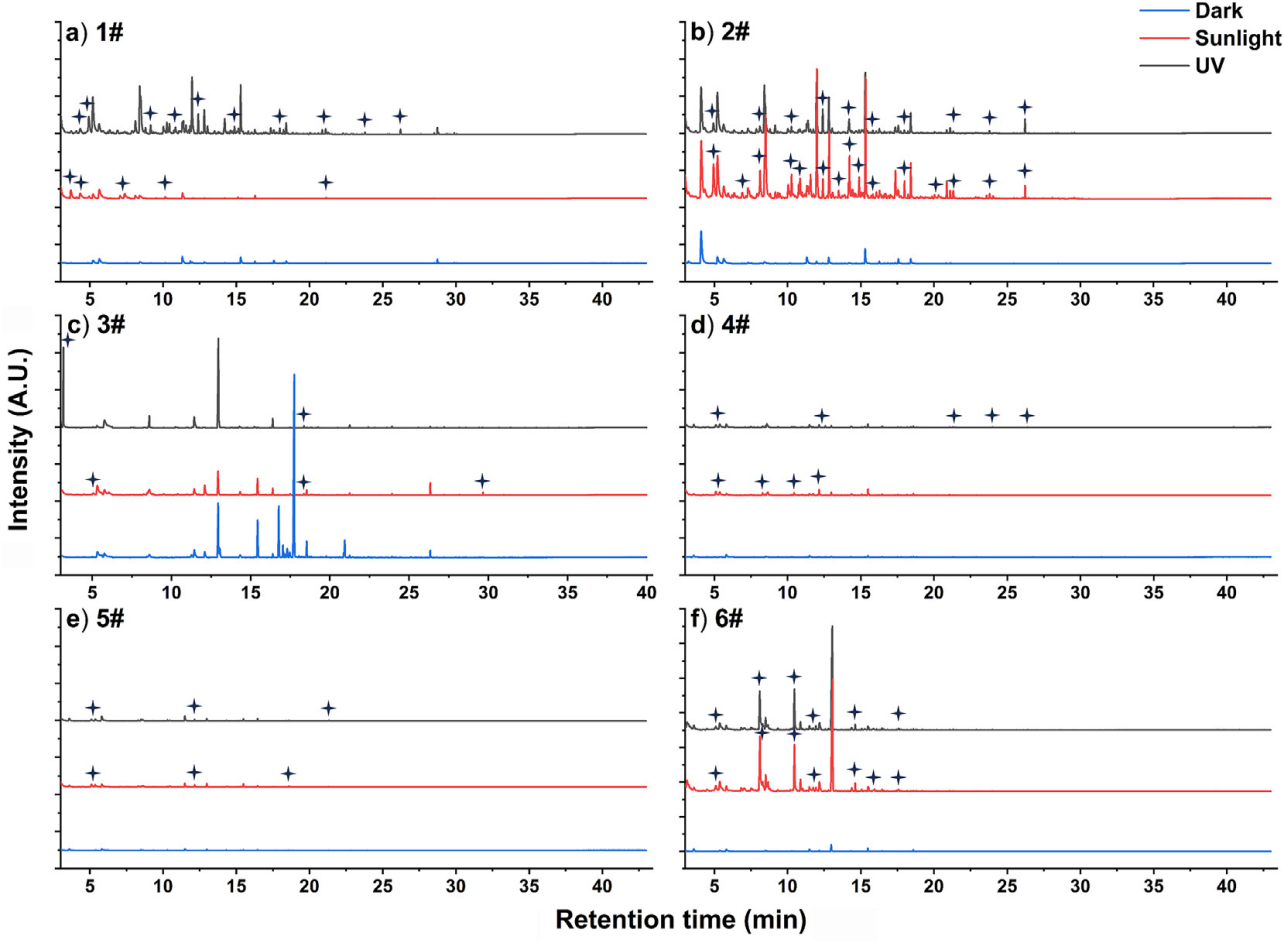
Total ion chromatogram of VOCs from bottles after short-term treatments. UV-A irradiation intensity: 5.6 W/m^2^, reaction time 12 h per day; Sunlight irradiation dose: 5.6 W/m^2^, irradiation time 12 h per day. Short-term experiment is conducted for 1 d, while long-term experiment is conducted for 7 d. Asterisk Mark indicates new peaks detected from the treated samples, which were not observed in the dark samples but could be observed in the treated samples. Only parts of the candidate peaks with high intensities were marked, but all these candidate peaks were analyzed.

The total ion chromatograms (TIC) of VOCs released from pristine, solar-treated, and UVA-treated containers are presented in Fig. 2. Notably, containers 1# and 2# exhibited abundant VOC generation after short-term UV-A irradiation, followed by container 6#, while the remaining three containers produced fewer products. The peaks mentioned herein represent new peaks that were not detected in samples stored under dark conditions but emerged in light-treated samples. It is crucial to note that these peaks have only undergone preliminary screening by mass spectrometry software, and further screening has not been performed, rendering them candidate product peaks.

The TIC peaks of the containers treated by solar and UV-A treatment were higher than those of the pristine containers. For example, the pristine 1# container displayed 28 peaks, whereas, after short-term solar and UV-A treatment, the number of peaks reached 37 and 81, respectively. Furthermore, the VOCs released from the 1# container after short-term UV-A treatment exceeded those released after solar treatment. The 2# container exhibited a higher number of VOC peaks after solar exposure (99 peaks), while the pristine and UV-A-treated conditions had relatively fewer peaks (32 and 81 peaks, respectively). Additionally, the VOCs generated by the 3# container after solar exposure were greater than those following UV-A irradiation, suggesting a higher release of VOCs from the 3# container induced by sunlight. These TIC results provided initial evidence indicating distinct characteristics of VOCs generated from different containers under UV-A and solar irradiation.

Long-term treatments demonstrated that plastic containers released more VOCs, as indicated by the detection of new peaks in the TIC results (Fig. S2). Specifically, the number of peaks in 1# container reached 97 after long-term solar treatment, more than double the number observed after short-term treatment. Similar changes in peak numbers were observed in the 3# container compared to the 1# container. Furthermore, in comparison to the short-term UV-A treatment, the number of peaks in the 1# container increased from 81 to 105 after long-term UV-A irradiation, and the number of the 2# container increased from 81 to 97. Additionally, new peaks appeared in the TIC results for containers 4#, 5#, and 6#. Notably, after long-term solar exposure, the release of VOCs decreased in the 6# container (27 peaks) compared to the short-term solar treatment (59 peaks). However, these findings indicated that the containers released a greater number of VOC products. A more comprehensive screening of VOC products based on the TIC results is warranted.

### 3.2 Non-targeted analysis of VOCs during short-term exposure

Only some of the above-observed peaks were identified as VOC candidates (Tables S4–S9). To prevent redundancy, VOCs are indicated by their CAS number when referred to for the second time in the subsequent section.

All the containers stored in the dark emitted small amounts of VOCs (Table 1). After being kept in the dark for 1 day, each container exhibited fewer than 15 species of observed VOCs (Table 1). Only 3# container released 14 species of VOCs. This observation aligned with the weak TIC intensities observed for all containers in Section 3.1. No peaks that could be attributed to additives were observed [24]. These results suggested that storing bottled water in the dark could be a safer storage method.

**Table 1:** Number of VOCs candidates from containers.

	Short-term treatment			Long-term treatment		
	Dark	UV-A	Sunlight	Dark	UV-A	Sunlight
1#	6	22	17	8	35	19
2#	3	28	30	10	32	32
3#	14	10	18	13	21	46
4#	3	10	14	0	19	15
5#	0	10	8	0	11	24
6#	2	19	12	3	37	6

After short-term UV-A or solar treatments, the release of VOCs from all containers increased (Fig. 2, Tables S4–S9), suggesting that both treatments induced the generation of various VOCs from containers. The composition patterns of VOCs were similar among different containers, likely due to their common PET material. Particularly, containers from the same brand exhibited relatively similar VOC compositions under different irradiations. The dominant VOCs included alkanes, alkenes, alcohols, aldehydes, carboxylic acids, ketones, esters, and other small molecule VOCs ranging from C3 to C12.

**Fig. 3:**
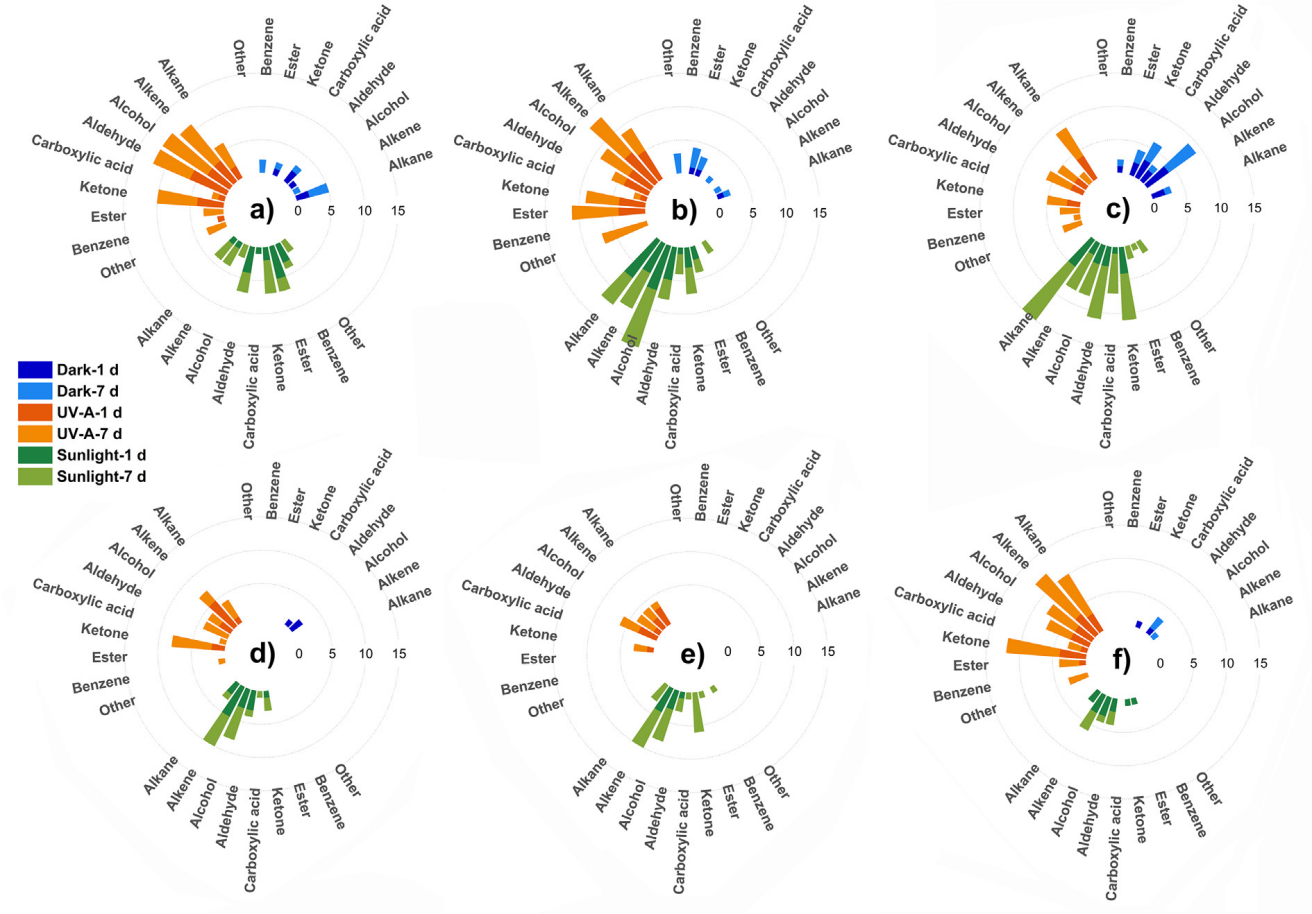
Evolutions patterns of VOCs category after different treatments. (a–f) #1–#6 bottles.

Taking the 1# container as an example, a total of 17 and 22 species of VOCs were released after solar and UV-A irradiation, respectively (Table 1). A large number of oxygen-containing VOCs were detected, suggesting their generation during the degradation and oxidation of the PET backbone under irradiation (Fig. 3). When the PET skeleton was exposed to UV-A irradiation, chemical changes occurred, including bond breaking and reconstruction. Small molecule fragments could detach from the PET skeleton, resulting in the generation of VOCs. These VOCs can further react with oxygen in the air, leading to the formation of oxygenated VOCs. For instance, short-chain small molecules, such as acetic acid (64-19-7), pentanal (110-62-3), 5-hexen-3-one (24253-30-3), and 1-hexanol (111-27-3), might be produced through the oxidation of the alkane structure in the PET skeleton. The release of VOCs from the 2# and 3# containers followed a similar pattern, whereas the other three containers exhibited lower VOC release (Fig. 3).

Significant differences were observed in the composition of VOCs between the samples stored in the dark and those subjected to irradiation. In the case of the 1# container, only 3 out of the 22 VOCs detected in the short-term UV-A treated sample, as well as 2 out of the 17 VOCs released in the short-term sunlight-treated sample, were presented in the pristine sample (Table S4). This indicated that the majority of the detected VOCs were generated after irradiation. Moreover, the VOCs generated through different irradiation treatments significantly differed. For example, only 4 VOCs were detected in both treatments for the 1# container. Similar results were observed for the other containers, demonstrating distinct degradation mechanisms between UV-A and solar treatments.

Differences were also observed in the oxidative VOCs generated during UV-A and solar irradiation. Taking the 1# container as an example, no alcohol was detected after short-term sunlight irradiation. In contrast, four alcohols were detected after short-term UV-A treatment, including linear chains, branched chains, and carbon ring structures (Table S4). However, the oxidation products produced by the 3# container after solar irradiation contained carboxylic acids, whereas no traces of carboxylic acids were found after UV-A treatment (Table S6). The 6# container exhibited more oxidative products after UV-A irradiation than after solar treatment, with some of the oxidation products having benzene rings and oxygen-containing heterocycles, while the oxidation products after solar irradiation lacked ring structures (Table S9). For the other three containers (2#, 4#, and 5#), similar amounts of oxidative VOCs were detected following both UV-A and solar irradiation. This can be attributed to the combination of heating and sunlight in the field, while UV-A treatment was conducted at room temperature. Furthermore, differences in container stability and additives may also contribute to these variations. Containers with poor stability are more susceptible to oxidation and may produce more and more complex oxidation products. Different additives may affect the degree of oxidation of PET molecules or the stability of oxidation products. The presence of abundant oxidative products indicated the occurrence of complex degradation reactions.

Previous studies have predominantly focused on the analysis of dissolved pollutants in bottled water released from containers, with limited consideration given to VOCs. Our findings indicated a substantial release of VOCs from plastic containers after light irradiation, which represented a significant new discovery. This highlighted the importance of considering VOCs as a potential risk factor associated with plastic container usage. Furthermore, previous studies have identified UV-A irradiation as the primary factor contributing to the degradation of PET containers in sunlight. However, our VOC results revealed notable differences in the VOCs generated under UV-A and solar irradiation. This suggested that not only UV-A but also visible light irradiation might contribute to the degradation of containers and the subsequent release of VOCs. This novel perspective significantly enhanced our understanding of the intricate degradation mechanisms involved in plastic containers.

### 3.3 Non-targeted analysis of VOCs during long-term exposure

In general, the containers treated by long-term irradiation released more VOCs compared to those exposed to short-term irradiation. For example, after long-term solar treatment, the 3# container showed an increase in the number of released VOCs from 18 to 46 (Table 1). In addition to the alkanes, alcohols, aldehydes, and ketones detected in the short-term samples, the presence of esters and benzene homologues in the long-term samples was also discovered. After the long-term UV-A treatment, the 3# container released 21 VOCs, approximately twice the number released in the short-term UV-A treatment, with three new types of substances (olefins, esters, and benzene homomers) identified (Fig. 3, Table S6). Notably, while short-term UV-A irradiation only resulted in VOCs with linear and branched chains from the 3# container, long-term UV-A treatment led to the appearance of new substances containing benzene and carbon rings. The aromatic organics could originate from the breakdown products of the PET structure or from the plasticizers added to the bottle. The carbon ring formation may occur as a result of PET chain scission after UV-A irradiation, where carbons at different positions on the molecular chain contain lone pairs of electrons that can react with each other to form a ring structure.

Furthermore, the VOCs produced by the same container following long-term sunlight and UV-A treatment also exhibited significant differences. Container 5# released 24 types of VOCs after long-term sunlight exposure (Table 1), while only 11 types were observed after long-term UV-A irradiation (Table S8). This discrepancy can be attributed to the fact that sunlight reaching the Earth's surface contains both UV-A and visible light radiation, which can induce PET degradation at multiple locations compared to UV-A alone, resulting in the generation of a wider range of VOCs. Despite all six containers being made of PET, the destruction-induced light irradiation could differ due to variations in their production processes and the addition of different additives, leading to diverse VOC generation patterns.

The duration of irradiation also played a significant role in shaping the composition of VOCs. A comparison between the long-term and short-term data revealed that the majority of VOCs differed even for the same container. Among the VOCs generated after long-term treatment, 24% (1#), 17% (2#), 13% (3#), 15% (4#), 12% (5#), and 1% (6#) of VOCs were detected in the short-term samples. This finding suggested a substantial change in VOC composition over time, indicating that UV-A and sunlight radiation continuously deteriorated the containers and led to the generation of increasingly complex VOCs.

### 3.4 Sources and identification

The safety of drinking water is of paramount importance, necessitating a comprehensive understanding of the sources and toxicological information of various VOCs. To gain insight into the sources of VOCs, a general screening was conducted, resulting in the identification of a wide range of compounds that can be categorized into 10 plastic-related compounds: (a) hydrocarbon, (b) fragrance (e.g., perfumers, flavor), (c) plasticizers, (d) raw material, (e) intermediates/monomers, (f) solvents, (g) dehydrating agent, (h) adhesive, (i) fuel and (j) plastic-related compounds with unknown source [25]. It should be noted that compounds categorized as (j) may be derived from the degradation products of plastics, although some compounds could originate from other sources. Therefore, in this article, compounds of type (j) will be referred to as “other reaction products”.

**Fig. 4:**
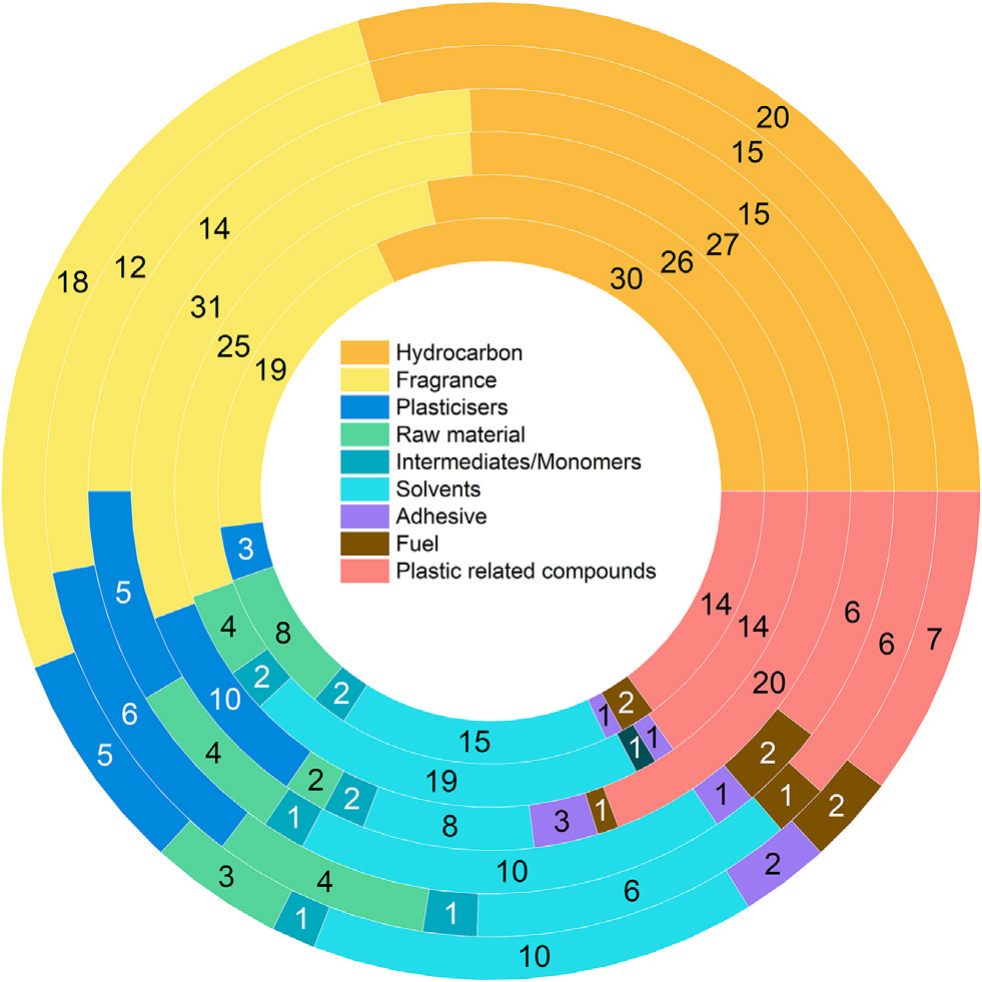
Source of VOCs. The long-term and short-term data were integrated.

The VOCs detected in this study can be broadly categorized into several groups: hydrocarbons (133 species), including straight-chain, branched-chain and cyclic alkanes, alkenes, halides, and aromatic hydrocarbons; fragrances (n = 119); solvents (n = 68); plasticizers (n = 29); raw materials (n = 25); intermediates/monomers (n = 9); fuels (n = 8); adhesives (n = 8) (Fig. 4). Additionally, there were 67 other reaction products identified. Further details for each VOC are provided in Tables S10–S15 and are discussed in more depth below.

Hydrocarbons can be categorized into various groups, including saturated hydrocarbons (such as straight-chain, substituted, and cyclic alkanes), unsaturated hydrocarbons, halogenated hydrocarbons, and aromatic hydrocarbons. During the screening process, a significant number of alkanes with carbon chain lengths ranging from C-6 to C-17 were identified, encompassing both short-chain and medium-chain compounds. Among the straight-chain and substituted alkanes, the most frequently detected compounds in the six containers were dodecane (112-40-3, n = 12), pentadecane (629-62-9, n = 15), hexadecane (544-76-3, n = 16), and heptadecane (629-78-7, n = 17). Linear alkanes and iso-alkanes are commonly derived from paraffin wax, widely used as an external lubricant in PET and other polymers. These external lubricants facilitate smooth polymer movement over various surfaces, including processing equipment. Alkanes, such as hexane, are also utilized as solvents. Short-chain alkenes with carbon chain lengths ranging from C-6 to C-14 were identified. These alkenes serve as starting compounds for various additives and polymers and can also be formed as by-products during olefin polymerization. Aromatic hydrocarbons, including benzene, were also detected. Benzene (71-43-2) is a raw material for dyes and synthetic detergents, and its derivatives are used in the production of dimethyl terephthalate, an essential component in PET manufacturing.

Fragrances are commonly utilized in various consumer products, including food and beverages, to impart specific scents or flavors. These compounds can be added as additives or lubricants to enhance the material properties or processability of products, and can possibly migrate into PET containers during the manufacturing process. Notably, 1#, 2#, and 3# containers generated more fragrances.

Solvents have been widely used in various industrial processes for dissolving or dispersing substances. In the production of PET containers, solvents play a crucial role in the pre-polymerization reaction and PET molding stages. Ideally, solvents should not persist in the final product. However, achieving complete conversion of solvents in industrial production is challenging. These solvents could be released upon exposure to sunlight irradiation. Notably, most of the detected plasticizers exhibited a notable presence in the samples that had been exposed to sunlight, but were rarely detected in the dark samples. Among them, 143-08-8, 111-27-3, 1-octanol (111-87-5), and 1-dodecanol (112-53-8) were the most dominant species. The level of plasticizers in plastic can amount up to 50% by weight. Thus, when plastic comes into contact with moisture, a considerable amount of water can leach out, and the plasticizers can volatilize into the air.

The remaining raw materials, intermediates/monomers, adhesives, and fuels are chemicals that can be incorporated into the PET production process. These additions can undergo direct evaporation from the container due to photo-stimulation or can cause the breakage of the polymer chain as a result of photo-stimulation, leading to subsequent reactions involving intermediates.

### 3.5 Toxicology analysis

**Fig. 5:**
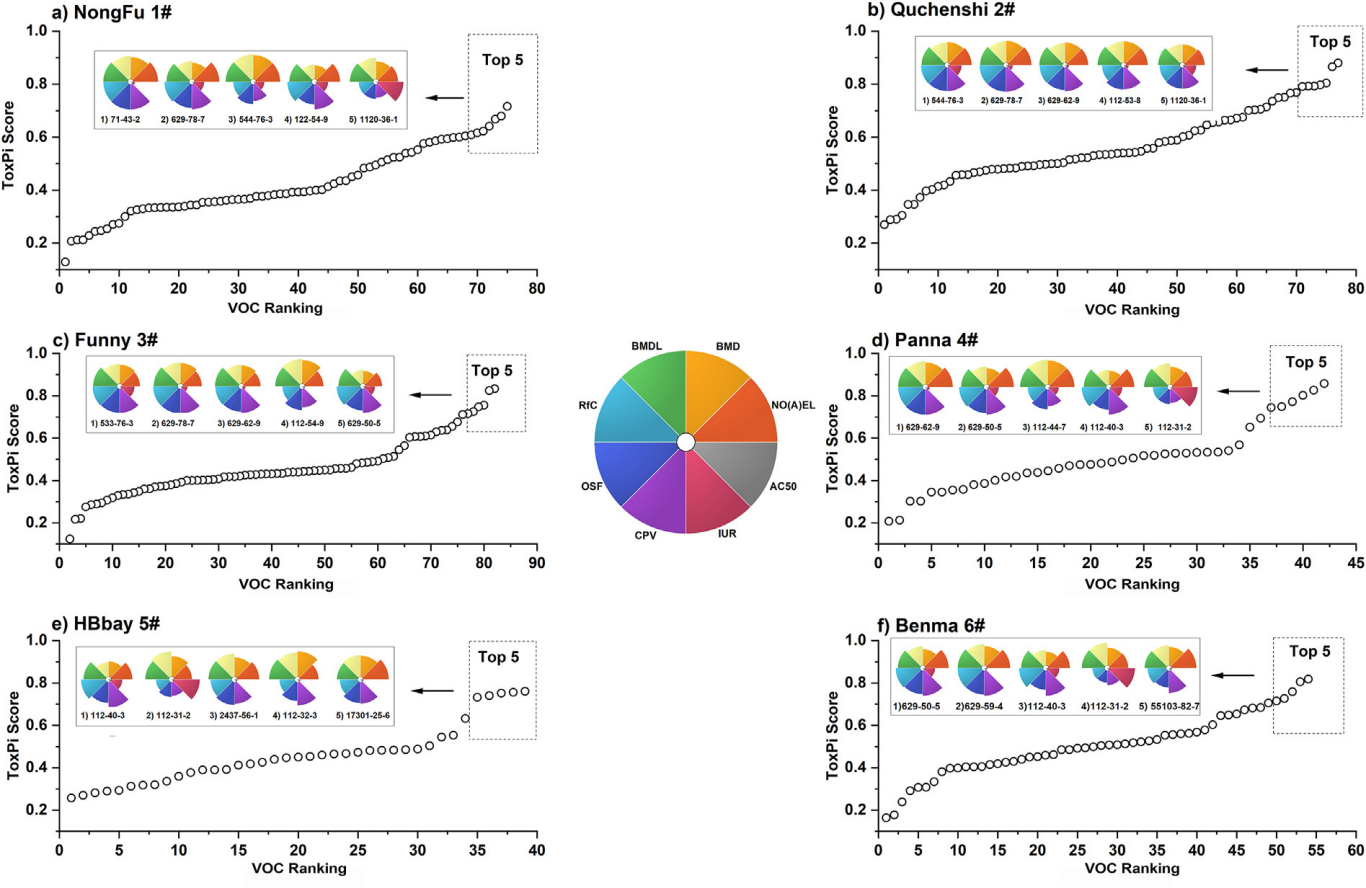
Toxicological prioritization schemes. (a) ToxPi score ranking of VOCs from 1# container, (b) ToxPi score ranking of VOCs from 2# container. For definitions of each scheme, refer to Section 2.7. Each slice represents an independent parameter, such as assays or analyses. The pie charts of top five toxic VOCs (with their CAS numbers) obtained from toxicological prioritization are presented in each graph. Individual slices represent data for the corresponding parameter. The distance between the outer edge of each slice and the center is the normalized value of the parameter. The angle occupied by a slice represents the weight of its parameter. Parameters include reference dose no observed adverse effect level [NO(A)EL, −log_10_ mg/(kg·day)], reference dose benchmark dose [BMD, −log_10_ mg/(kg·day)], reference dose benchmark dose lower limit [BMDL, −log_10_ mg/(kg·day)], reference concentration (RfC, −log_10_ mg/m^3^), oral slope factor [OSF, log_10_ risk per Mol/(kg·day)], cancer potency value [CPV, log_10_ risk per mg/(kg·day)], inhalation unit risk (IUR, log_10_ risk per μg/m^3^) from CTV, half-maximal concentration (AC50, −log_10_ μM) from ToxCast.

The emission of volatile organic compounds (VOCs) into the atmosphere is intricately linked to industrial production, transportation, and various other processes. Prolonged exposure to hazardous VOCs can lead to health issues, particularly those associated with carbonyl compounds and aromatic compounds, which exert the most pronounced influence on both the environment and human health. Notably, persistent exposure to acetone may impact the nervous system, formaldehyde has been linked to nasopharyngeal cancer, and excessive benzene exposure can result in fatality [26]. Consequently, it is imperative to conduct screening and predictive assessments for the identification of highly toxic VOCs. The ToxPi definition and ranking results are presented in Fig. 5. Rankings of 75, 77, 81, 42, 39, and 54 VOCs were conducted for six containers, respectively. CTV predictor covered the human toxicity values of all VOCs, while ToxCast only provided the assay data of 51%, 47%, 51%, 49%, 43%, and 48% VOCs for these samples, respectively. The limited availability of half-maximal concentration (AC50) values can be attributed to certain tests providing qualitative or semi-quantitative results instead of quantitative values, as well as the ongoing development of the database.

For 1# container, the top five toxic VOCs were methyl benzene (108-88-3), 544-76-3, 629-78-7, dodecanal (112-54-9), and 1-tetradecene (1120-36-1) (Fig. 5, Table S16). Among these VOCs, 108-88-3 is a common chemical agent that is irritating to the respiratory tract. Thus, it exhibits high values in oral slope factor (OSF) and cancer potency value (CPV), indicating a significant risk to human health through oral exposure, particularly in relation to cancer. Furthermore, it showed elevated values in inhalation unit risk (IUR), suggesting increased health risks associated with inhalation exposure. Compound 108-88-3, which is commonly used as an intermediate in chemical synthesis, was detected in the sample following short-term solar irradiation. Initial exposure to solvents can result in solvent intake through inhalation, dermal absorption, and oral ingestion. Epidemiological studies have provided substantial evidence linking high-level 108-88-3 exposures to an increased risk of acute myelogenous leukemia in humans.

The other four VOCs were short-chain alkanes, alkenes, and aldehydes, which could be generated from the decomposition of PET. They were found after long-term UV-A treatment, exhibiting high reference dose, no observed adverse effect level [NO(A)EL], benchmark dose (BMD), benchmark dose lower limit (BMDL), and reference concentration (RfC) values. This indicated that these four VOCs posed high noncancer health risks to humans through oral exposure. Importantly, all of them are straight-chain compounds. Medium-chain alkanes and alkenes of this nature are commonly utilized in various industries and applications, including solvents, lubricants, and starting materials for chemical synthesis. Although they may exhibit low acute toxicity, prolonged or excessive exposure to these substances can have potential implications for human health and the environment. Compound 112-54-9, a short-chain aldehyde, is frequently employed as an additive or fragrance in diverse sectors such as cosmetics, detergents, and the food industry. It is worth considering the possibility that 112-54-9 could originate from the exposure of drinking water to light. Notably, El-Maghrabey et al. [27] have suggested that chlorination, ozonation, and UV exposure of drinking water for sterilization purposes could lead to the abundant formation of aldehydes. In conclusion, these findings implied that long-term UV-A treatment has the potential to induce the generation of toxic VOCs from the 1# container.

For 2# container, the top five toxic VOCs were 544-76-3, 629-78-7, n-pentadecane (629-62-9), 112-53-8, and 1120-36-1. Among these, 544-76-3, 629-78-7, and 1120-36-1 were once again detected in the samples from the 2# container. These five VOCs exhibited similar toxicity profiles, with high values observed for all indicators except AC50, indicating their significant overall toxicity to humans among the detected VOCs from the 2# container. This suggested a potential inhalation toxicity risk for humans. It should be noted that these compounds were oxidized compounds detected in long-term treatments of the container samples, suggesting that the further oxidation products generated after UV-A and solar irradiation could possess high toxicity.

For the other four containers, most of the top five toxic VOCs also exhibited elevated values in nearly all parameters, with the exception of AC50. However, decanal (112-31-2), which was detected in 4#, 5#, and 6# containers, displayed a high AC50 value. Table 1 lists the five VOCs with the highest potential toxicity for each container, and it is worth noting that several VOCs appeared repeatedly. Compounds 544-76-3, 629-78-7, 112-54-9, 112-31-2, 629-62-9 and tridecane (629-50-5) were consistently detected. These recurring VOCs accounted for 18 of the 30 VOCs on the list, indicating the presence of common toxic VOCs across different containers. Despite variations in the sources of raw materials used in the containers, their molecular structures are all composed of PET, which may explain the similarities in the toxic VOCs and their toxicity profiles. In general, it was observed that various potentially toxic VOCs were generated under light conditions, particularly following long-term irradiation.

### 3.6 Yields of toxic VOCs

According to the ToxPi data, standard samples of the top five VOCs of each container were purchased for quantitative detection. The results are shown in Table S17. Among the top five VOCs from 1# container, 108-88-3 was only detected in the short-term sunlight sample, with yields at 0.62 ng/g. Two alkanes, 544-76-3 and 629-78-7, were only detected in the long-term UV-A-sample, with yields of 0.18 ng/g and 0.11 ng/g, respectively. Compound 1120-36-1 had a yield of 0.24 ng/g. The only oxygenated VOC, 112-54-9, yielded 0.23 ng/g. Except for benzene, the yields of these VOCs were low.

The occurrence probabilities and yields of the top five VOCs in the 2# container were higher compared to those in the 1# container. Among them, the toxic 544-76-3 was detected in samples exposed to short-term UV-A, short-term sunlight, and long-term sunlight. The highest yield of 544-76-3, reaching 0.75 ng/g, was observed after long-term sunlight treatment. The yields of other VOCs were also notably high. Similarly, the top five VOCs detected in the 3# container showed a high occurrence probability, with alkanes and oxygenated alkanes being the predominant compounds. After long-term sunlight treatment, the yield of 544-76-3 reached 0.59 ng/g. Additionally, in the long-term sunlight samples, the yield of 629-78-7 reached 0.68 ng/g, while 112-54-9 reached 0.79 ng/g. These VOCs exhibited relatively high yields and were consistently present in the long-term irradiation experimental groups.

The yields of the top five VOCs from both the 4# and 5# containers were all below 0.2 ng/g. However, the yields of toxic VOCs from the 6# container were higher. In particular, the yields of the alkanes 629-50-5 and 112-40-3 in the long-term UV-A experimental group reached 0.32 ng/g and 0.45 ng/g, respectively. Overall, while the yields of toxic VOCs from the 2# and 3# containers were relatively high, these levels were still low. Considering the mass of a plastic container (16–25 g) as the source, the yields of toxic VOCs would only amount to several ng per container.

### 3.7 Yields of toxic VOCs in the existence of water matrices

The selected research objects were the samples with high VOC yields, specifically the 2# and 6# containers. VOC release was measured after UV-A and solar irradiation using different solutions as matrices. It was observed that the background substances in the solution matrices influenced the VOC yields. In the case of the 2# container, the top 5 VOCs were not detected in the dark sample. Following long-term UV-A treatment, the yields of the top 5 VOCs in deionized water exhibited a similar pattern to the previous results in deionized water. However, in the presence of the mineral solution, the yields of various VOCs were significantly increased. For instance, the yield of 112-53-8 increased from 0.46 ng/g to 0.65 ng/g. Conversely, the yields of VOCs decreased in the soda solution. The yield of 112-53-8 decreased to 0.16 ng/g, and the other VOCs were not detected. In the case of solar treatments, the variations in VOC yields among different matrices followed a similar pattern to those observed in UV-A treatments, with enhanced yields in the mineral solution and inhibited yields in the soda solution.

For the 6# container, VOCs were only detected after long-term UV-A treatment and were absent in long-term solar treatment. Similar enhancement and inhibition effects were observed in the presence of the mineral solution and soda solution, respectively. The mineral solution contained Zn^2+^ and Sr^2+^ ions, which likely facilitated degradation and oxidation reactions under light irradiation, leading to an increased degradation rate of the containers. Consequently, more VOCs were generated in the mineral solution. On the other hand, carbonate ions in the soda solution inhibited degradation and oxidation processes, and also acted as quenchers for free radicals. This property significantly inhibited free radical reactions, even terminating them. Therefore, in the presence of soda water, the aging and degradation of containers were inhibited, resulting in reduced yields of VOCs. Based on these experimental results, bottled soda water may be a safer option.

## 4 Conclusion

Our findings demonstrated that light exposure induced the emission of a diverse range of VOCs from various containers, encompassing alkanes, alkenes, alcohols, aldehydes, carboxylic acids, aromatics, etc. Notably, we observed significant variations in the VOC composition released by different containers under different irradiation conditions. Moreover, containers exposed to prolonged irradiation exhibited distinct VOC profiles compared to those exposed to shorter durations. The yield of toxic VOCs ranged from 0.02 ng/g to 0.79 ng/g following extended irradiation periods. Crucially, our investigation revealed that the type of water matrix had a discernible impact on VOC generation. Mineral water was found to enhance VOC yields, while soda water exhibited an inhibitory effect. Considering the average weight of a container (approximately 20 g), the amount of VOCs volatilized from a single container was only a few nanograms. Consequently, even after long-term exposure, opening and consuming water from a bottled container poses minimal health risks to humans.

## CRediT authorship contribution statement

R.J.L.: writing–original draft, writing–reviewing and editing, software; Z.A.Q.L., J.Z.: data curation, writing–original draft preparation; X.N.W.: software, supervision; Z.Y.T.: software, validation; H.S.O.: ideas, conceptualization, methodology, writing–original draft, writing–reviewing and editing, project administration, funding acquisition.

## Declaration of competing interests

The authors declare that they have no known competing financial interests or personal relationships that could have appeared to influence the work reported in this paper.
