# Supplementary material for: Characterizing the photodegradation-induced release of volatile organic compounds from bottled water containers
Source: Eco Environ Health. 2024 Feb 8;3(2):145–53. doi: 10.1016/j.eehl.2024.01.005 (PMC11021827; doi:10.1016/j.eehl.2024.01.005)
Supplement: Multimedia component 1 [file mmc1.pdf]

Supplementary Material

Characterizing the Photodegradation-Induced Release of Volatile Organic Compounds from Bottled Water Containers

Ruijuan Liu [a, b], Zhianqi Liao [a, c], Jing Zheng [b], Xinni Wu [a, c], Zongyi Tan [a, c], Huase Ou* [a, c]

[a] *Guangdong Key Laboratory of Environmental Pollution and Health, School of Environment, Jinan University, Guangzhou 511443, China*

[b] *State Environmental Protection Key Laboratory of Environmental Pollution Health Risk Assessment, South China Institute of Environmental Sciences, Ministry of Ecology and Environment, Guangzhou 510655, China*

[c] *Key Laboratory of Philosophy and Social Science in Guangdong Province of Community of Life for Man and Nature, Jinan University, Guangzhou 511443, China*

Text S1 Toxicity prediction ranking

Conditional Toxicity Value (CTV) predictor provides reference dose no observed adverse effect level (NO(A)EL, -log_{10} mg kg^{-1} day^{-1}), reference dose benchmark dose (BMD, -log_{10} mg kg^{-1} day^{-1}), reference dose benchmark dose lower limit (BMDL, -log_{10} mg kg^{-1} day^{-1}), reference concentration (RfC, -log_{10} mg $(m^3)^{-1}$), oral slope factor (OSF, log_{10} risk per Mol/(kg·day)), cancer potency value (CPV, log_{10} risk per mg kg^{-1} day^{-1}), inhalation unit risk (IUR, log_{10} risk per μg $(m^3)^{-1}$). ToxCast screening library provides half-maximal concentration (AC50, -log_{10} μM). Specifically, the above 8 parameters were imported into ToxPi, with each parameter corresponded to a slice and assigned a weight of 12.5%.

Table S1. Detailed information of bottle water

Container	Brand	Volume (mL)	Source area	Water type
#1	Nongfu Spring	550	China	Spring water
#2	Watsons	500	China	Distilled water
#3	Fannybay	500	Canada	Purified by artesian water
#4	Panna	500	Italy	Spring water
#5	Hbay	500	New Zealand	Spring water
#6	Benma	500	Japan	Artesian water

Table S2. Characteristic parameters of solution matrices

Parameters	Deionized water	Mineral water (mg L^{-1})	Soda water
pH	6.45	6.12	8.4
DOC	0.05	0.04	0.06
Zn^{2+}	0	0.56	0
Sr^{2+}	0	0.67	0
HCO_3^-	0	0	0.49

Table S3. Recovery of high toxic VOCs detected by SPME-GCMS.

Compound	Recovery ratio (%)
Benzene	96.2±0.9
Hexadecane	95.6±0.6
Heptadecane	96.8±2.4
Dodecanal	102.7±1.2
Ethylbenzene	105.6±0.8
Pentadecane	102.8±0.7
1-Dodecanol	97.1±2.6
1-Tetradecene	99.9±1.8
Tridecane	92.9±1.9
Undecanal	107.8±2.1
Dodecane	106.9±3.1
Decanal	98.7±0.5
Formic acid, octyl ester	95.8±0.9
Undecane, 2,8-dimethyl-	93.0±0.8
Tetradecane	97.3±1.9
2-Dodecene, 2-methyl-	106.8±3.2

Experiments were conducted in triplicate.

Table S4. Mass spectrum information of VOCs released from the 1# bottle after different detected reaction

Compound	Formula	CAS	RT[1]	RI_{exp}[2]	RI_{lib}[3]	Detected reaction						m/z
						Dark-1 d	UV-A-1 d	Sunlight-1 d	Dark-7 d	UV-A-7 d	Sunlight-7 d	
Acetone	C_3H_6O	67-64-1	1.725	496.01	486	√	√	√		√	√	43
1-Pentene, 2-methyl-	C_6H_{12}	763-29-1	2.071	566.93	580				√	√		85
2-Butanone	C_4H_8O	78-93-3	2.107	574.44	598						√	43
n-Hexane	C_6H_{14}	110-54-3	2.109	574.85	600	√			√			57
Acetic acid	$C_2H_4O_2$	64-19-7	2.184	590.06	610		√	√		√		43
Benzene	C_6H_6	71-43-2	2.612	648.34	654			√				78
1-Heptene	C_7H_{14}	592-76-7	2.901	684.99	685		√					57
1-Butanol, 2-methyl-, (S)-	$C_5H_{12}O$	1565-80-6	2.910	686.16	697					√		41
Pentanal	$C_5H_{10}O$	110-62-3	3.013	699.27	699		√	√	√			44
3-Pentanone, 2-methyl-	$C_6H_{12}O$	565-69-5	3.022	700.17	745						√	57
n-Propyl acetate	$C_5H_{10}O_2$	109-60-4	3.243	710.14	708			√		√		43
Methyl Isobutyl Ketone	$C_6H_{12}O$	108-10-1	3.708	731.91	735				√			43
1-Heptene, 6-methyl-	C_8H_{16}	5026-76-6	4.078	748.98	749						√	56
sec-Butyl acetate	$C_6H_{12}O_2$	105-46-4	4.093	749.70	760			√				43
Toluene	C_7H_8	108-88-3	4.333	760.80	763			√				91
5-Hexen-3-one	$C_6H_{10}O$	24253-30-3	4.391	763.49	744			√				29
2-Pentanone, 4,4-dimethyl-	$C_7H_{14}O$	590-50-1	4.662	776.03	758		√			√		43
1-Octene	C_8H_{16}	111-66-0	4.941	788.93	789			√				43
Hexanal	$C_6H_{12}O$	66-25-1	5.218	801.15	800		√	√		√		44
Acetic acid, butyl ester	$C_6H_{12}O_2$	123-86-4	5.620	813.73	812			√		√		43
3-Heptene, 4-methyl-	C_8H_{16}	4485-16-9	5.933	823.30	802					√		55
Cyclopentanol, 2-methyl-, cis-	$C_6H_{12}O$	25144-05-2	6.560	842.75	849					√		57
2-Butanol, 3-methyl-, acetate	$C_7H_{14}O_2$	5343-96-4	6.668	846.10	830			√				43
Ethylbenzene	C_8H_{10}	100-41-4	7.064	858.37	855			√				107
1-Octene, 6-methyl-	C_9H_{18}	13151-10-5	7.151	861.06	853					√		55
Octane, 4-methyl-	C_9H_{20}	2216-34-4	7.160	861.32	863			√				127
Hexanal, 5-methyl-	$C_7H_{14}O$	1860-39-5	7.285	865.21	841		√			√	√	43
1-Hexanol	$C_6H_{14}O$	111-27-3	7.379	868.10	868		√			√		56
1-Butanol, 3-methyl-, acetate	$C_7H_{14}O_2$	123-92-2	7.649	876.47	876			√				43
Hexanal, 4-methyl-	$C_7H_{14}O$	41065-97-8	7.662	876.90	889					√		70
3-Hexanone, 5-methyl-	$C_7H_{14}O$	623-56-3	7.919	884.83	855					√		57
Nonane	C_9H_{20}	111-84-2	8.414	900.17	900						√	43
Hexanal, 3-methyl-	$C_7H_{14}O$	19269-28-4	8.491	902.39	910			√				70

2-Propenoic acid, anhydride	$C_6H_6O_3$	2051-76-5	8.800	911.33	901						√	55
3-Heptanol, 4-methyl-	$C_8H_{18}O$	14979-39-6	9.159	921.67	915					√		59
meso-3,4-Hexanediol	$C_6H_{14}O_2$	22520-39-4	9.165	921.85	942				√			59
2,5-Hexanedione	$C_6H_{10}O_2$	110-13-4	9.293	925.56	921						√	43
2-Octene, 2,6-dimethyl-	$C_{10}H_{20}$	4057-42-5	9.657	936.07	936						√	69
2(5H)-Furanone, 5,5-dimethyl-	$C_6H_8O_2$	20019-64-1	10.036	947.02	952		√			√	√	97
2-Heptanone, 6-methyl-	$C_8H_{16}O$	928-68-7	10.274	953.90	956					√		43
1-Heptanol	$C_7H_{16}O$	111-70-6	10.845	970.36	970		√					70
1-Hexanol, 2,2-dimethyl-	$C_8H_{18}O$	2370-13-0	11.052	976.36	975						√	57
1-Decene	$C_{10}H_{20}$	872-05-9	11.559	991.00	989		√			√		41
Decane	$C_{10}H_{22}$	124-18-5	11.862	999.73	1,000						√	85
Octanal	$C_8H_{16}O$	124-13-0	11.974	1,003.12	1,003	√		√		√		43
Benzene, 1-methyl-3-(1-methylethyl)-	$C_{10}H_{14}$	535-77-3	12.489	1,018.67	1,023		√				√	93
1-Hexanol, 2-ethyl-	$C_8H_{18}O$	104-76-7	12.830	1,028.95	1,030	√	√			√		57
2(3H)-Furanone, 5-ethenyldihydro-5-methyl-	$C_7H_{10}O_2$	1073-11-6	13.045	1,035.43	1,043		√			√		111
2-Octen-1-ol	$C_8H_{16}O$	22104-78-5	13.482	1,048.63	1,066					√		57
Nonane, 2,8-dimethyl-4-methylene-	$C_{12}H_{24}$	7323-15-1	13.722	1,055.89	1,053							56
1-Octanol	$C_8H_{18}O$	111-87-5	14.221	1,070.94	1,071					√		56
Formic acid, 2-ethylhexyl ester	$C_9H_{18}O_2$	5460-45-7	14.435	1,077.41	1,092						√	57
1-Undecene	$C_{11}H_{22}$	821-95-4	14.878	1,090.77	1,091		√					71
Undecane	$C_{11}H_{24}$	1120-21-4	15.179	1,099.88	1,100				√		√	43
Nonanal	$C_9H_{18}O$	124-19-6	15.303	1,103.87	1,104	√	√					41
2-Undecene, 9-methyl-, (Z)-	$C_{12}H_{24}$	74630-45-8	17.070	1,161.11	1,158					√	√	70
2-Nonen-1-ol, (E)-	$C_9H_{18}O$	31502-14-4	17.357	1,170.41	1,176				√			57
Levomenthol	$C_{10}H_{20}O$	2216-51-5	17.565	1,177.12	1,175		√			√		71
Undecane, 3,5-dimethyl-	$C_{13}H_{28}$	17312-81-1	18.236	1,198.87	1,207					√		57
Dodecane	$C_{12}H_{26}$	112-40-3	18.238	1,198.94	1,200	√						57
Decanal	$C_{10}H_{20}O$	112-31-2	18.408	1,204.78	1,206					√	√	41
3-Isopropylbenzaldehyde	$C_{10}H_{12}O$	34246-57-6	18.854	1,220.30	1,234		√					133
Tetrahydrogeranyl formate	$C_{11}H_{22}O_2$	68214-06-2	19.853	1,255.07	1,251					√		55
2-Decenal, (E)-	$C_{10}H_{18}O$	3913-81-3	20.034	1,261.36	1,263		√					41
Undecane, 2,3-dimethyl-	$C_{13}H_{28}$	17312-77-5	20.082	1,263.04	1,251					√		43
1-Tridecene	$C_{13}H_{26}$	2437-56-1	20.874	1,290.61	1,292		√			√		97
Tridecane	$C_{13}H_{28}$	629-50-5	21.097	1,298.36	1,300		√					43
Undecanal	$C_{11}H_{22}O$	112-44-7	21.305	1,305.99	1,307						√	43
Dodecane, 2,7,10-trimethyl-	$C_{15}H_{32}$	74645-98-0	21.673	1,319.73	1,320				√			57
2-Methyl-1-undecanol	$C_{12}H_{26}O$	10522-26-6	23.582	1,390.98	1,393						√	57

1-Tetradecene	$C_{14}H_{28}$	1120-36-1	23.584	1,391.03	1,392		√	41
Dodecanal	$C_{12}H_{24}O$	112-54-9	24.043	1,409.07	1,409		√	41
Pentadecane, 7-methyl-	$C_{16}H_{34}$	6165-40-8	26.217	1,499.16	1,541	√		57
Hexadecane	$C_{16}H_{34}$	544-76-3	28.074	1,600.04	1,600		√	85
Heptadecane	$C_{17}H_{36}$	629-78-7	29.579	1,700.41	1,700		√	57

[1] RT: Retention time.

[2] RI_{exp}: Retention index obtained from experiment. It calculation is based on formula:

$$RI = 100n + 100(RT_x - RT_n)/(RT_{n+1} - RT_n)$$

where, n is the carbon atom number of the nalkane outflowed before target compound, RT_x is the retention time of target compound, RT_n and RT_{n+1} are the retention times of nalkanes outflowed before and after target compounds.

[3] RI_{lib}: Retention index in the NIST library.

Table S5. Mass spectrum information of VOCs released from the 2# bottle after different detected reaction

Compound	Formula	CAS	RT[1]	RI_{exp}[2]	RI_{lib}[3]	Detected reaction						m/z
						Dark-1 d	UV-A-1 d	Sunlight-1 d	Dark-7 d	UV-A-7 d	Sunlight-7 d	
Acetone	C_3H_6O	67-64-1	1.728	496.63	486	√	√	√		√	√	43
Formic acid	CH_2O_2	64-18-6	1.755	502.08	526						√	29
1-Pentene, 2-methyl-	C_6H_{12}	763-29-1	2.075	567.87	580			√		√		85
n-Hexane	C_6H_{14}	110-54-3	2.105	574.01	600	√						57
Ethyl Acetate	$C_4H_8O_2$	141-78-6	2.182	589.68	612	√						43
Acetic acid	$C_2H_4O_2$	64-19-7	2.188	590.93	610		√	√			√	43
1-Heptene	C_7H_{14}	592-76-7	2.900	684.88	685		√			√		57
Pentanal	$C_5H_{10}O$	110-62-3	3.010	698.85	699		√					44
Butanal, 2,2-dimethyl-	$C_6H_{12}O$	2094-75-9	3.019	700.00	722				√			43
n-Propyl acetate	$C_5H_{10}O_2$	109-60-4	3.214	709.05	708		√	√	√	√	√	43
Furan, 2-methoxy-	$C_5H_6O_2$	25414-22-6	3.464	720.59	737				√			83
Methyl Isobutyl Ketone	$C_6H_{12}O$	108-10-1	3.696	731.34	735				√			43
sec-Butyl acetate	$C_6H_{12}O_2$	105-46-4	4.100	750.01	760				√	√		43
2-Pentanone, 4,4-dimethyl-	$C_7H_{14}O$	590-50-1	4.655	775.71	758					√	√	43
1-Octene	C_8H_{16}	111-66-0	4.950	789.33	789		√		√			43
Acetic acid, butyl ester	$C_6H_{12}O_2$	123-86-4	5.613	813.40	812				√	√		43
2-Hexene, 2,3-dimethyl-	C_8H_{16}	7145-20-2	5.933	823.31	789			√				83
1-Methylpentyl cyclopropane	C_9H_{18}	6976-28-9	6.891	853.01	854						√	56
1-Octene, 6-methyl-	C_9H_{18}	13151-10-5	7.141	860.76	853			√		√	√	55
4-Isopropoxy-2-butanone	$C_7H_{14}O_2$	32541-58-5	7.283	865.14	865						√	43
1-Methoxy-2-propyl acetate	$C_6H_{12}O_3$	108-65-6	7.287	865.27	870		√					43
Hexanal, 4-methyl-	$C_7H_{14}O$	41065-97-8	7.649	876.49	889							70
3-Heptanone	$C_7H_{14}O$	106-35-4	7.913	884.67	887					√	√	57
2-Heptanone	$C_7H_{14}O$	110-43-0	8.041	888.62	891					√		43
1-Nonene	C_9H_{18}	124-11-8	8.080	889.82	889		√			√		71
1-Octene, 2,6-dimethyl-	$C_{10}H_{20}$	6874-29-9	8.708	908.65	918						√	56
3-Heptanol, 4-methyl-	$C_8H_{18}O$	14979-39-6	9.145	921.28	915					√	√	59

Propanoic acid, anhydride	$C_6H_{10}O_3$	123-62-6	9.150	921.43	921			√	√		57
3-Hexanol, 4-ethyl-	$C_8H_{18}O$	19780-44-0	9.163	921.79	915		√				59
4-Nonene	C_9H_{18}	2198-23-4	9.336	926.79	924	√			√		55
Heptanal	$C_7H_{14}O$	111-71-7	9.452	930.14	901					√	44
1-Hexanol, 5-methyl-	$C_7H_{16}O$	627-98-5	9.652	935.91	930				√	√	43
2(5H)-Furanone, 5,5-dimethyl-	$C_6H_8O_2$	20019-64-1	10.039	947.10	952	√			√		97
2-Heptanone, 6-methyl-	$C_8H_{16}O$	928-68-7	10.278	953.99	956		√				43
1-Heptanol	$C_7H_{16}O$	111-70-6	10.843	970.31	970	√				√	70
3-Pentenoic acid, 4-methyl-	$C_6H_{10}O_2$	504-85-8	11.144	979.01	1011				√	√	43
1-Hepten-6-one, 2-methyl-	$C_8H_{14}O$	10408-15-8	11.352	985.03	966			√			43
1-Decene	$C_{10}H_{20}$	872-05-9	11.562	991.07	989		√			√	41
2-Hepten-4-one, 2-methyl-	$C_8H_{14}O$	22319-24-0	11.756	996.67	938	√					89
Octane, 3,4,5,6-tetramethyl-	$C_{12}H_{26}$	62185-21-1	11.858	999.62	958					√	57
Octanal	$C_8H_{16}O$	124-13-0	11.978	1,003.23	1003	√				√	43
Ether, 6-methylheptyl vinyl	$C_{10}H_{20}O$	10573-35-0	12.400	1,015.98	1017				√		57
1-Hexanol, 2-ethyl-	$C_8H_{18}O$	104-76-7	12.844	1,029.38	1030	√	√				57
2-Octen-1-ol	$C_8H_{16}O$	22104-78-5	13.488	1,048.82	1066		√				57
4-Decene, 3-methyl-, (E)-	$C_{11}H_{22}$	62338-47-0	13.792	1,058.01	1059	√				√	55
Acetophenone	C_8H_8O	98-86-2	14.017	1,064.79	1065	√	√				105
1-Octanol	$C_8H_{18}O$	111-87-5	14.231	1,071.24	1071		√		√		56
Formic acid, 2-ethylhexyl ester	$C_9H_{18}O_2$	5460-45-7	14.441	1,077.59	1092	√			√	√	57
1-Undecene	$C_{11}H_{22}$	821-95-4	14.877	1,090.74	1091	√			√		71
Undecane	$C_{11}H_{24}$	1120-21-4	15.163	1,099.39	1100	√	√		√		43
Nonanal	$C_9H_{18}O$	124-19-6	15.307	1,103.99	1104	√	√				41
(S)-(+)-6-Methyl-1-octanol	$C_9H_{20}O$	110453-78-6	16.491	1,142.36	1094					√	55
Acetic acid, 2-ethylhexyl ester	$C_{10}H_{20}O_2$	103-09-3	16.606	1,146.08	1129	√			√		43
1-Octanol, 2,7-dimethyl-	$C_{10}H_{22}O$	15250-22-3	16.881	1,154.98	1130					√	56
2-Nonenal, (E)-	$C_9H_{16}O$	18829-56-6	17.025	1,159.63	1162		√				43
1-Undecene, 9-methyl-	$C_{12}H_{24}$	74630-41-4	17.067	1,161.01	1152					√	70
1-Nonanol	$C_9H_{20}O$	143-08-8	17.369	1,170.79	1173		√		√	√	56
Levomenthol	$C_{10}H_{20}O$	2216-51-5	17.564	1,177.09	1175					√	71

Dodecane	$C_{12}H_{26}$	112-40-3	18.236	1,198.87	1200	√	√			√	57
Decane, 6-ethyl-2-methyl-	$C_{13}H_{28}$	62108-21-8	18.242	1,199.06	1185			√			57
Decanal	$C_{10}H_{20}O$	112-31-2	18.418	1,205.11	1206		√		√		41
Tetrahydrogeranyl formate	$C_{11}H_{22}O_2$	68214-06-2	19.863	1,255.43	1251		√		√		55
2-Decenal, (E)-	$C_{10}H_{18}O$	3913-81-3	20.039	1,261.52	1263	√					41
1-Decanol	$C_{10}H_{22}O$	112-30-1	20.312	1,271.04	1273		√				55
1-Tridecene	$C_{13}H_{26}$	2437-56-1	20.864	1,290.25	1292		√			√	97
Dodecane, 2,6,11-trimethyl-	$C_{15}H_{32}$	31295-56-4	21.097	1,298.37	1275				√		43
Tridecane	$C_{13}H_{28}$	629-50-5	21.126	1,299.35	1300	√	√				43
2-Undecenal	$C_{11}H_{20}O$	2463-77-6	22.856	1,363.87	1367		√				41
1-Undecanol	$C_{11}H_{24}O$	112-42-5	23.579	1,390.85	1371	√	√			√	55
1-Tetradecene	$C_{14}H_{28}$	1120-36-1	23.583	1,391.00	1392				√		41
Tetradecane	$C_{14}H_{30}$	629-59-4	23.798	1,399.03	1400	√	√			√	57
Dodecanal	$C_{12}H_{24}O$	112-54-9	24.041	1,408.99	1409		√		√	√	41
1,9-Nonanediol	$C_9H_{20}O_2$	3937-56-2	24.042	1,409.03	1401	√					68
1-Dodecanol	$C_{12}H_{26}O$	112-53-8	26.060	1,492.65	1473				√	√	55
Pentadecane	$C_{15}H_{32}$	629-62-9	26.223	1,499.42	1500		√		√		71
Hexadecane	$C_{16}H_{34}$	544-76-3	28.077	1,600.22	1600		√		√	√	85
Heptadecane	$C_{17}H_{36}$	629-78-7	29.571	1,699.83	1700	√	√				57

[1] RT: Retention time.

[2] RI_{exp}: Retention index obtained from experiment. It calculation is based on formula:

$$RI = 100n + 100(RT_x - RT_n)/(RT_{n+1} - RT_n)$$

where, n is the carbon atom number of the nalkane outflowed before target compound, RT_x is the retention time of target compound, RT_n and RT_{n+1} are the retention times of nalkanes outflowed before and after target compounds.

[3] RI_{lib}: Retention index in the NIST library.

Table S6. Mass spectrum information of VOCs released from the 3# bottle after different detected reaction

Compound	Formula	CAS	RT[1]	RI_{exp}[2]	RI_{lib}[3]	Detected reaction						m/z
						Dark-1 d	UV-A-1 d	Sunlight-1 d	Dark-7 d	UV-A-7 d	Sunlight-7 d	
2-Butanol, 2,3-dimethyl-	$C_6H_{14}O$	594-60-5	3.662	729.78	720					√		59
1-Pentanol	$C_5H_{12}O$	71-41-0	3.716	732.25	765					√		55
Butane, 2,2,3,3-tetramethyl-	C_8H_{18}	594-82-1	3.783	735.37	726					√		57
Pentane, 2,2,3-trimethyl-	C_8H_{18}	564-02-3	3.79	735.67	737		√					43
Propanoic acid, 2-methyl-	$C_4H_8O_2$	79-31-2	4.02	746.42	774	√		√	√		√	43
Pentanal, 2-methyl-	$C_6H_{12}O$	123-15-9	4.051	747.76	759					√		43
Toluene	C_7H_8	108-88-3	4.428	765.21	763					√	√	91
Pentane, 2,2,4,4-tetramethyl-	C_9H_{20}	1070-87-7	4.480	767.59	773						√	56
1-Octene	C_8H_{16}	111-66-0	5.053	794.10	789					√		55
1-Heptene, 3-methyl-	C_8H_{16}	4810-09-7	5.06	794.39	747			√				55
Octane	C_8H_{18}	111-65-9	5.290	803.39	800			√		√	√	43
2-Pentanone, 3-ethyl-	$C_7H_{14}O$	6137-03-7	6.036	826.52	838					√		43
2-Hexanone, 3-methyl-	$C_7H_{14}O$	2550-21-2	6.04	826.77	844			√				43
Butanoic acid, 3-methyl-	$C_5H_{10}O_2$	503-74-2	6.521	841.53	863	√			√		√	60
Butanoic acid, 2-methyl-	$C_5H_{10}O_2$	116-53-0	6.80	850.33	861	√					√	74
Heptane, 2,5,5-trimethyl-	$C_{10}H_{22}$	1189-99-7	7.41	869.18	892			√				71
Formic acid, hexyl ester	$C_7H_{14}O_2$	629-33-4	7.497	871.76	914					√		56
Hexanal, 5-methyl-	$C_7H_{14}O$	1860-39-5	7.497	871.77	841						√	43
Benzene, 1,3-dimethyl-	C_8H_{10}	108-38-3	7.51	872.19	866	√			√			91
Hexanal, 4-methyl-	$C_7H_{14}O$	41065-97-8	7.784	880.68	889						√	70
3-Heptanone	$C_7H_{14}O$	106-35-4	8.040	888.60	887						√	57
Pentanoic acid	$C_5H_{10}O_2$	109-52-4	8.069	889.49	904						√	60
1-Nonene	C_9H_{18}	124-11-8	8.24	894.76	889			√			√	56
Cyclohexanone	$C_6H_{10}O$	108-94-1	8.327	897.48	894					√		55
Nonane	C_9H_{20}	111-84-2	8.53	903.51	900		√					43
Hexane, 2,2,3,3-tetramethyl-	$C_{10}H_{22}$	13475-81-5	8.539	903.79	929						√	43
Hexanal, 3-methyl-	$C_7H_{14}O$	19269-28-4	8.60	905.68	910	√						70
Heptanal	$C_7H_{14}O$	111-71-7	8.615	905.96	901						√	70

meso-3,4-Hexanediol	$C_6H_{14}O_2$	22520-39-4	9.270	924.89	942				√			59
4-Nonene	C_9H_{18}	2198-23-4	9.473	930.75	924						√	69
1-Hydroxy-2-pentanone	$C_5H_{10}O_2$	64502-89-2	9.51	931.72	897		√					43
Ethanone, 1-cyclopentyl-	$C_7H_{12}O$	6004-60-0	9.51	931.83	941			√				71
2(5H)-Furanone, 5,5-dimethyl-	$C_6H_8O_2$	20019-64-1	10.149	950.28	952						√	97
2-Heptanone, 6-methyl-	$C_8H_{16}O$	928-68-7	10.391	957.26	956						√	43
Hexanal, 2-ethyl-	$C_8H_{16}O$	123-05-7	10.394	957.35	956			√		√		72
4-Oxohex-2-enal	$C_6H_8O_2$	20697-55-6	10.524	961.10	957						√	55
Heptane, 3,3,4-trimethyl-	$C_{10}H_{22}$	20278-87-9	10.93	972.88	937		√					71
1-Heptanol	$C_7H_{16}O$	111-70-6	10.966	973.88	970			√	√		√	70
Hexanoic acid	$C_6H_{12}O_2$	142-62-1	11.248	982.02	990				√			60
5-Hepten-2-one, 6-methyl-	$C_8H_{14}O$	110-93-0	11.45	987.89	986	√			√			43
Hexanal, 2,2-dimethyl-	$C_8H_{16}O$	996-12-3	11.472	988.47	993					√		57
4-Penten-1-ol, propanoate	$C_8H_{14}O_2$	30563-30-5	11.590	991.89	974					√		57
1-Decene	$C_{10}H_{20}$	872-05-9	11.695	994.92	989						√	56
Decane	$C_{10}H_{22}$	124-18-5	11.984	1003.42	1000					√		57
3,4-Hexanedione, 2,2,5-trimethyl-	$C_9H_{16}O_2$	20633-03-8	11.99	1003.55	1039		√	√				57
Nonane, 4-methyl-	$C_{10}H_{22}$	17301-94-9	11.992	1003.66	961						√	57
Octanal	$C_8H_{16}O$	124-13-0	12.10	1006.89	1003			√			√	43
1-Decene, 8-methyl-	$C_{11}H_{22}$	61142-79-8	12.382	1015.42	1056					√		70
Pentane, 1-(1-butenyloxy)-, (E)-	$C_9H_{18}O$	54004-25-0	12.529	1019.88	1000						√	43
1-Hexanol, 2-ethyl-	$C_8H_{18}O$	104-76-7	12.94	1032.42	1030	√	√		√			57
1-Propanol, 2-(2-hydroxypropoxy)-	$C_6H_{14}O_3$	106-62-7	13.368	1045.19	1046	√		√	√		√	59
2,7-Octanedione	$C_8H_{14}O_2$	1626-09-1	13.503	1049.27	1088						√	43
4-Decene, 3-methyl-, (E)-	$C_{11}H_{22}$	62338-47-0	13.914	1061.68	1059						√	55
Acetophenone	C_8H_8O	98-86-2	14.124	1068.02	1065				√	√	√	105
1-Octanol	$C_8H_{18}O$	111-87-5	14.33	1074.14	1071	√	√				√	55
3-Nonanone	$C_9H_{18}O$	925-78-0	14.805	1088.58	1090						√	72
4-Decene, 4-methyl-, (E)-	$C_{11}H_{22}$	60366-66-7	15.011	1094.80	1100						√	70
6-Hepten-3-one, 5-hydroxy-4,6-dimethyl-	$C_9H_{16}O_2$	62338-59-4	15.287	1103.35	1117						√	57
Nonanal	$C_9H_{18}O$	124-19-6	15.429	1107.95	1104		√	√		√	√	57

2-Nonen-1-ol	$C_9H_{18}O$	22104-79-6	15.435	1108.14	1105				√			57
Hexanoic acid, 2-ethyl-	$C_8H_{16}O_2$	149-57-5	15.778	1119.25	1123						√	88
Formic acid, octyl ester	$C_9H_{18}O_2$	112-32-3	16.181	1132.30	1114						√	56
Undecane, 5-methylene-	$C_{12}H_{24}$	5698-48-6	16.405	1139.55	1181						√	56
l-Menthone	$C_{10}H_{18}O$	14073-97-3	17.03	1159.96	1148	√						112
1-Nonanol	$C_9H_{20}O$	143-08-8	17.48	1174.30	1173			√		√		56
Levomenthol	$C_{10}H_{20}O$	2216-51-5	17.75	1183.03	1175	√						95
Dodecane	$C_{12}H_{26}$	112-40-3	18.361	1203.14	1200						√	57
Butanoic acid, 4-hexenyl ester, (Z)-	$C_{10}H_{18}O_2$	69727-41-9	18.535	1209.20	1203					√		67
Decanal	$C_{10}H_{20}O$	112-31-2	18.536	1209.21	1206	√	√		√		√	43
1-(2,3-Dimethylphenyl)ethanone	$C_{10}H_{12}O$	2142-71-4	18.98	1224.85	1255			√				133
2-Ethylhexyl acrylate	$C_{11}H_{20}O_2$	103-11-7	19.131	1229.94	1220						√	55
1-Decanol	$C_{10}H_{22}O$	112-30-1	19.198	1232.27	1273						√	55
Nonane, 5-methyl-5-propyl-	$C_{13}H_{28}$	62238-11-3	20.210	1267.48	1229						√	57
Nonanoic acid	$C_9H_{18}O_2$	112-05-0	20.296	1270.49	1273						√	60
Tridecane	$C_{13}H_{28}$	629-50-5	21.23	1303.29	1300			√			√	57
1-Undecanol	$C_{11}H_{24}O$	112-42-5	23.186	1376.21	1371						√	56
Tetradecane	$C_{14}H_{30}$	629-59-4	23.92	1404.11	1400	√	√	√	√			57
Dodecanal	$C_{12}H_{24}O$	112-54-9	24.155	1413.73	1409						√	57
Pentadecane	$C_{15}H_{32}$	629-62-9	26.332	1505.15	1500					√	√	57
Hexadecane	$C_{16}H_{34}$	544-76-3	28.166	1606.19	1600	√		√		√	√	57
Heptadecane	$C_{17}H_{36}$	629-78-7	29.665	1707.02	1700						√	57

[1] RT: Retention time.

[2] RI_{exp}: Retention index obtained from experiment. It calculation is based on formula:

$$RI = 100n + 100(RT_x - RT_n)/(RT_{n+1} - RT_n)$$

where, n is the carbon atom number of the nalkane outflowed before target compound, RT_x is the retention time of target compound, RT_n and RT_{n+1} are the retention times of nalkanes outflowed before and after target compounds.

[3] RI_{lib}: Retention index in the NIST library.

Table S7. Mass spectrum information of VOCs released from the 4# bottle after different detected reaction

Compound	Formula	CAS	RT[1]	RI_{exp}[2]	RI_{lib}[3]	Detected reaction						m/z
						Dark-1 d	UV-A-1 d	Sunlight-1 d	Dark-7 d	UV-A-7 d	Sunlight-7 d	
Isopropyl Alcohol	C_3H_8O	67-63-0	1.715	494.00	486	√						45
Acetic acid	$C_2H_4O_2$	64-19-7	2.092	571.30	610						√	43
Propanal, 2-methyl-	C_4H_8O	78-84-2	2.163	585.91	552			√				43
1-Heptene	C_7H_{14}	592-76-7	3.019	699.99	685			√		√	√	57
Acetylacetone	$C_5H_8O_2$	123-54-6	3.834	737.71	783					√		43
Methyl Isobutyl Ketone	$C_6H_{12}O$	108-10-1	3.835	737.75	735			√			√	43
Propanoic acid, 2-methyl-	$C_4H_8O_2$	79-31-2	4.063	748.29	774					√		43
1-Heptene, 6-methyl-	C_8H_{16}	5026-76-6	4.214	755.31	749						√	56
1-Hexene, 3,3,5-trimethyl-	C_9H_{18}	13427-43-5	4.224	755.74	757		√	√				65
Toluene	C_7H_8	108-88-3	4.473	767.27	763					√		91
Pentane, 2,2,3-trimethyl-	C_8H_{18}	564-02-3	4.528	769.83	737			√				57
2-Pentanone, 4,4-dimethyl-	$C_7H_{14}O$	590-50-1	4.832	783.90	758					√		43
1-Octene	C_8H_{16}	111-66-0	5.093	795.98	789		√				√	43
Octane	C_8H_{18}	111-65-9	5.340	804.96	800			√			√	43
1-Pentanol, 3-methyl-	$C_6H_{14}O$	589-35-5	7.075	858.71	838		√			√		56
1-Hepten-3-one	$C_7H_{12}O$	2918-13-0	7.331	866.63	881					√		55
2-Pentanone, 3-ethyl-3-methyl-	$C_8H_{16}O$	19780-65-5	7.390	868.46	868						√	43
3-Heptanone	$C_7H_{14}O$	106-35-4	8.085	889.98	887					√		57
1-Nonene	C_9H_{18}	124-11-8	8.293	896.44	889		√			√	√	71
1-Hexanol, 3-methyl-	$C_7H_{16}O$	13231-81-7	8.296	896.52	896			√				63
Heptanal	$C_7H_{14}O$	111-71-7	8.653	907.06	901					√		44
2(5H)-Furanone, 5,5-dimethyl-	$C_6H_8O_2$	20019-64-1	10.199	951.71	952					√		97
2-Heptanone, 6-methyl-	$C_8H_{16}O$	928-68-7	10.462	959.31	956		√			√		43
Benzaldehyde	C_7H_6O	100-52-7	10.689	965.86	962					√		106
1-Heptanol	$C_7H_{16}O$	111-70-6	11.015	975.27	970						√	70
1-Heptanol, 6-methyl-	$C_8H_{18}O$	1653-40-3	11.734	996.04	995						√	41

1-Decene	$C_{10}H_{20}$	872-05-9	11.753	996.60	989		√	√				41
3,4-Hexanedione, 2,2,5-trimethyl-	$C_9H_{16}O_2$	20633-03-8	12.046	1,005.29	1039		√					43
Decane	$C_{10}H_{22}$	124-18-5	12.050	1,005.40	1000					√		85
Octanal	$C_8H_{16}O$	124-13-0	12.138	1,008.07	1003						√	43
1-Hexanol, 2-ethyl-	$C_8H_{18}O$	104-76-7	12.984	1,033.60	1030	√		√				57
1-Octanol	$C_8H_{18}O$	111-87-5	14.367	1,075.37	1071			√			√	56
(S)-(+)-6-Methyl-1-octanol	$C_9H_{20}O$	110453-78-6	15.054	1,096.10	1094						√	55
Nonanal	$C_9H_{18}O$	124-19-6	15.486	1,109.81	1104			√				41
1-Nonanol	$C_9H_{20}O$	143-08-8	17.519	1,175.66	1173		√			√	√	56
5-Undecene, 6-methyl-	$C_{12}H_{24}$	83687-45-0	18.168	1,196.67	1199		√	√			√	41
Dodecane	$C_{12}H_{26}$	112-40-3	18.424	1,205.34	1200					√		57
Decanal	$C_{10}H_{20}O$	112-31-2	18.592	1,211.18	1206	√		√		√		41
1-Tridecene	$C_{13}H_{26}$	2437-56-1	21.061	1,297.10	1292			√				97
Tridecane	$C_{13}H_{28}$	629-50-5	21.286	1,305.27	1300		√					43
Undecanal	$C_{11}H_{22}O$	112-44-7	21.496	1,313.14	1307					√		43
Pentadecane	$C_{15}H_{32}$	629-62-9	26.394	1,508.53	1500					√		71

[1] RT: Retention time.

[2] RI_{exp}: Retention index obtained from experiment. It calculation is based on formula:

$$RI = 100n + 100(RT_x - RT_n)/(RT_{n+1} - RT_n)$$

where, n is the carbon atom number of the nalkane outflowed before target compound, RT_x is the retention time of target compound, RT_n and RT_{n+1} are the retention times of nalkanes outflowed before and after target compounds.

[3] RI_{lib}: Retention index in the NIST library.

Table S8. Mass spectrum information of VOCs released from the 5# bottle after different detected reaction

Compound	Formula	CAS	RT[1]	RI_{exp}[2]	RI_{lib}[3]	Detected reaction						m/z
						Dark-1 d	UV-A-1 d	Sunlight-1 d	Dark-7 d	UV-A-7 d	Sunlight-7 d	
Acetic acid	$C_2H_4O_2$	64-19-7	2.140	581.21	610						√	43
1-Hexene	C_6H_{12}	592-41-6	2.152	583.57	584			√				56
Propanal, 2-methyl-	C_4H_8O	78-84-2	2.169	587.06	552		√					43
3-Methyl-3-hexene	C_7H_{14}	3404-65-7	3.022	700.17	702		√	√		√	√	56
3-Pentanone, 2-methyl-	$C_6H_{12}O$	565-69-5	3.128	705.06	745						√	57
Acetylacetone	$C_5H_8O_2$	123-54-6	3.839	737.94	783						√	43
1-Penten-3-one, 2,4-dimethyl-	$C_7H_{12}O$	3212-68-8	4.221	755.59	756		√					69
1-Heptene, 6-methyl-	C_8H_{16}	5026-76-6	4.240	756.51	749						√	56
1-Hexene, 3,3,5-trimethyl-	C_9H_{18}	13427-43-5	4.252	757.05	757					√		65
1-Octene	C_8H_{16}	111-66-0	5.103	796.40	789			√			√	43
Octane	C_8H_{18}	111-65-9	5.364	805.68	800		√				√	43
Butanal, 3,3-dimethyl-2-oxo-, hemihydrate	$C_6H_{10}O_2$	4480-47-1	7.066	858.41	858		√					57
2-Pentanone, 3-ethyl-3-methyl-	$C_8H_{16}O$	19780-65-5	7.399	868.74	868						√	43
1-Hexanol	$C_6H_{14}O$	111-27-3	7.541	873.14	868						√	56
3-Heptanone	$C_7H_{14}O$	106-35-4	8.085	889.98	887					√	√	57
1-Hexanol, 3-methyl-	$C_7H_{16}O$	13231-81-7	8.294	896.47	896		√	√				63
1-Nonene	C_9H_{18}	124-11-8	8.295	896.50	889			√				71
3-Heptanol	$C_7H_{16}O$	589-82-2	8.494	902.49	877						√	59
Nonane	C_9H_{20}	111-84-2	8.599	905.52	900		√					43
Heptanal	$C_7H_{14}O$	111-71-7	8.651	907.00	901					√	√	44
2(5H)-Furanone, 5,5-dimethyl-	$C_6H_8O_2$	20019-64-1	10.195	951.61	952					√		97
Hexanal, 2-ethyl-	$C_8H_{16}O$	123-05-7	10.451	958.99	956			√				72
4-Nonene, 3-methyl-, (Z)-	$C_{10}H_{20}$	63830-69-3	10.722	966.82	959						√	55
1-Heptanol	$C_7H_{16}O$	111-70-6	11.011	975.16	970			√		√	√	70
1-Heptanol, 6-methyl-	$C_8H_{18}O$	1653-40-3	11.747	996.42	995					√		41
3-Decene	$C_{10}H_{20}$	19398-37-9	11.753	996.59	988		√					41

Octanal	$C_8H_{16}O$	124-13-0	12.151	1,008.46	1003				√		43
3,6-Heptanedione	$C_7H_{12}O_2$	1703-51-1	12.795	1,027.90	1041					√	43
1-Hexanol, 2-ethyl-	$C_8H_{18}O$	104-76-7	13.068	1,036.12	1030					√	57
2-Decene, 9-methyl-, (Z)-	$C_{11}H_{22}$	74630-24-3	13.911	1,061.59	1059					√	69
Acetophenone	C_8H_8O	98-86-2	14.173	1,069.51	1065					√	105
1-Octanol	$C_8H_{18}O$	111-87-5	14.378	1,075.69	1,071		√			√	56
Undecane	$C_{11}H_{24}$	1120-21-4	15.340	1,105.08	1100				√	√	43
Formic acid, octyl ester	$C_9H_{18}O_2$	112-32-3	16.233	1,134.00	1114					√	41
1-Nonanol	$C_9H_{20}O$	143-08-8	18.162	1,196.46	1173				√		56
Undecane, 2,8-dimethyl-	$C_{13}H_{28}$	17301-25-6	18.411	1,204.89	1,220	√					43
Dodecane	$C_{12}H_{26}$	112-40-3	18.415	1,205.02	1200					√	57
Decanal	$C_{10}H_{20}O$	112-31-2	18.585	1,210.95	1206	√			√	√	41
1-Tridecene	$C_{13}H_{26}$	2437-56-1	21.055	1,296.90	1292					√	97

[1] RT: Retention time.

[2] RI_{exp}: Retention index obtained from experiment. It calculation is based on formula:

$$RI = 100n + 100(RT_x - RT_n)/(RT_{n+1} - RT_n)$$

where, n is the carbon atom number of the nalkane outflowed before target compound, RT_x is the retention time of target compound, RT_n and RT_{n+1} are the retention times of nalkanes outflowed before and after target compounds.

[3] RI_{lib}: Retention index in the NIST library.

Table S9. Mass spectrum information of VOCs released from the 6# bottle after different detected reaction

Compound	Formula	CAS	RT[1]	RI_{exp}[2]	RI_{lib}[3]	Detected reaction						m/z
						Dark-1 d	UV-A-1 d	Sunlight-1 d	Dark-7 d	UV-A-7 d	Sunlight-7 d	
Isopropyl Alcohol	C_3H_8O	67-63-0	1.715	494.03	486		√		√			45
Butane, 2-methoxy-	$C_5H_{12}O$	6795-87-5	1.886	528.93	530		√					57
1-Pentene, 2-methyl-	C_6H_{12}	763-29-1	2.149	583.04	580					√	√	85
3-Methyl-3-hexene	C_7H_{14}	3404-65-7	3.022	700.16	702					√	√	41
2-Butanone, 3,3-dimethyl-	$C_6H_{12}O$	75-97-8	3.131	705.19	678		√					57
Methyl Isobutyl Ketone	$C_6H_{12}O$	108-10-1	3.819	737.01	735		√					43
Acetylacetone	$C_5H_8O_2$	123-54-6	3.827	737.37	783					√		43
1-Hexene, 3,3,5-trimethyl-	C_9H_{18}	13427-43-5	4.235	756.27	757		√					65
1-Heptene, 6-methyl-	C_8H_{16}	5026-76-6	4.252	757.04	749					√		56
1-Octene	C_8H_{16}	111-66-0	5.103	796.41	789			√		√		43
Hexanal	$C_6H_{12}O$	66-25-1	5.371	805.89	800	√	√					44
2-Hexene, 2,3-dimethyl-	C_8H_{16}	7145-20-2	6.105	828.64	789					√		83
Heptane, 2,3-dimethyl-	C_9H_{20}	3074-71-3	7.395	868.61	855					√		71
Pentane, 2,3,3,4-tetramethyl-	C_9H_{20}	16747-38-9	7.412	869.14	861		√					43
3-Heptanone	$C_7H_{14}O$	106-35-4	8.115	890.92	887		√	√		√		57
1-Nonene	C_9H_{18}	124-11-8	8.298	896.59	889		√					71
Hexanal, 3-methyl-	$C_7H_{14}O$	19269-28-4	8.658	907.21	910					√	√	70
1-Hexanol, 4-methyl-	$C_7H_{16}O$	818-49-5	8.795	911.17	953					√		70
2-Heptenol	$C_7H_{14}O$	33467-76-4	9.631	935.31	968					√		57
2H-Pyran-2-carboxaldehyde, 5,6-dihydro-	$C_6H_8O_2$	53897-26-0	9.749	938.72	940		√			√		83
2(5H)-Furanone, 5,5-dimethyl-	$C_6H_8O_2$	20019-64-1	10.205	951.88	952					√		97
Hexanal, 2-ethyl-	$C_8H_{16}O$	123-05-7	10.441	958.71	956				√		√	72
Benzaldehyde	C_7H_6O	100-52-7	10.690	965.91	962		√					106
1-Heptanol	$C_7H_{16}O$	111-70-6	11.013	975.23	970		√	√			√	70
3-Pentenoic acid, 4-methyl-	$C_6H_{10}O_2$	504-85-8	11.323	984.18	1011					√		43
5-Hepten-2-one, 6-methyl-	$C_8H_{14}O$	110-93-0	11.505	989.43	986	√						43

2-Octanone	$C_8H_{16}O$	111-13-7	11.680	994.47	990				√		58
1-Decene	$C_{10}H_{20}$	872-05-9	11.752	996.58	989		√		√	√	41
2-Hexenal, 2-ethyl-	$C_8H_{14}O$	645-62-5	11.930	1,001.77	999		√				55
Decane	$C_{10}H_{22}$	124-18-5	12.049	1,005.38	1000				√		85
Octanal	$C_8H_{16}O$	124-13-0	12.153	1,008.52	1003			√	√		43
3,6-Heptanedione	$C_7H_{12}O_2$	1703-51-1	12.781	1,027.47	1041				√		43
1-Hexanol, 2-ethyl-	$C_8H_{18}O$	104-76-7	13.058	1,035.82	1030	√	√		√		57
2-Decene, 6-methyl-, (Z)-	$C_{11}H_{22}$	74630-31-2	13.907	1,061.46	1059				√		43
4-Decene, 3-methyl-, (E)-	$C_{11}H_{22}$	62338-47-0	14.098	1,067.22	1059				√		55
Acetophenone	C_8H_8O	98-86-2	14.182	1,069.75	1065	√			√		105
1-Octanol	$C_8H_{18}O$	111-87-5	14.385	1,075.89	1071		√				56
Formic acid, 2-ethylhexyl ester	$C_9H_{18}O_2$	5460-45-7	14.622	1,083.04	1092	√	√		√		57
3-Nonanone	$C_9H_{18}O$	925-78-0	14.862	1,090.30	1090				√		43
1-Octanol, 2-methyl-	$C_9H_{20}O$	818-81-5	15.069	1,096.54	1094				√		57
1-Undecene	$C_{11}H_{22}$	821-95-4	15.069	1,096.55	1091	√	√				71
Undecane	$C_{11}H_{24}$	1120-21-4	15.341	1,105.12	1100		√		√		43
Nonanal	$C_9H_{18}O$	124-19-6	15.485	1,109.78	1110				√		41
Hexanoic acid, 2-ethyl-	$C_8H_{16}O_2$	149-57-5	15.878	1,122.48	1123	√					88
Formic acid, octyl ester	$C_9H_{18}O_2$	112-32-3	16.234	1,134.02	1114				√		41
Acetic acid, 2-ethylhexyl ester	$C_{10}H_{20}O_2$	103-09-3	16.791	1,152.08	1129				√		43
1-Nonanol	$C_9H_{20}O$	143-08-8	17.531	1,176.03	1173				√		56
5-Undecene, 6-methyl-	$C_{12}H_{24}$	83687-45-0	18.173	1,196.84	1199	√	√				55
Dodecane	$C_{12}H_{26}$	112-40-3	18.418	1,205.12	1200				√		57
Decanal	$C_{10}H_{20}O$	112-31-2	18.593	1,211.22	1206		√		√		41
Nonanoic acid	$C_9H_{18}O_2$	112-05-0	20.270	1,269.58	1273				√		117
2-Dodecene, 2-methyl-	$C_{13}H_{26}$	55103-82-7	21.059	1,297.02	1299				√		69
Tridecane	$C_{13}H_{28}$	629-50-5	21.289	1,305.39	1300	√			√		43
Tetradecane	$C_{14}H_{30}$	629-59-4	23.991	1,406.91	1400				√		57

[1] RT: Retention time.

[2] RI_{exp}: Retention index obtained from experiment. It calculation is based on formula:

$$RI = 100n + 100(RT_x - RT_n)/(RT_{n+1} - RT_n)$$

where, n is the carbon atom number of the nalkane outflowed before target compound, RT_x is the retention time of target compound, RT_n and RT_{n+1} are the retention times of nalkanes outflowed before and after target compounds.

[3] RI_{lib}: Retention index in the NIST library.

Table S10. Potential sources of VOCs released from the 1# bottle

Compound	Formula	CAS	Source	Detected reaction					
				Dark-1 d	UV-A-1 d	Sunlight-1 d	Dark-7 d	UV-A-7 d	Sunlight-7 d
Acetone	C_3H_6O	67-64-1	Solvents /Intermediates	√	√	√		√	√
1-Pentene, 2-methyl-	C_6H_{12}	763-29-1	Hydrocarbon				√	√	
2-Butanone	C_4H_8O	78-93-3	Solvents						√
n-Hexane	C_6H_{14}	110-54-3	Hydrocarbon	√			√		
Acetic acid	$C_2H_4O_2$	64-19-7	Raw material		√	√		√	
Benzene	C_6H_6	71-43-2	Hydrocarbon			√			
1-Heptene	C_7H_{14}	592-76-7	Hydrocarbon		√				
1-Butanol, 2-methyl-, (S)-	$C_5H_{12}O$	1565-80-6	Plastic related compounds					√	
Pentanal	$C_5H_{10}O$	110-62-3	Raw material		√	√	√		
3-Pentanone, 2-methyl-	$C_6H_{12}O$	565-69-5	Raw material						√
n-Propyl acetate	$C_5H_{10}O_2$	109-60-4	Fragrance			√		√	
Methyl Isobutyl Ketone	$C_6H_{12}O$	108-10-1	Adhesive/Solvents /Raw material				√		
1-Heptene, 6-methyl-	C_8H_{16}	5026-76-6	Hydrocarbon						√
sec-Butyl acetate	$C_6H_{12}O_2$	105-46-4	Solvents			√			
Toluene	C_7H_8	108-88-3	Hydrocarbon			√			
5-Hexen-3-one	$C_6H_{10}O$	24253-30-3	Plastic related compounds			√			
2-Pentanone, 4,4-dimethyl-	$C_7H_{14}O$	590-50-1	Solvents		√			√	
1-Octene	C_8H_{16}	111-66-0	Hydrocarbon/Plasticizers			√			
Hexanal	$C_6H_{12}O$	66-25-1	Fragrance		√	√		√	
Acetic acid, butyl ester	$C_6H_{12}O_2$	123-86-4	Solvents			√		√	
3-Heptene, 4-methyl-	C_8H_{16}	4485-16-9	Hydrocarbon					√	
Cyclopentanol, 2-methyl-, cis-	$C_6H_{12}O$	25144-05-2	Plastic related compounds					√	
2-Butanol, 3-methyl-, acetate	$C_7H_{14}O_2$	5343-96-4	Plastic related compounds			√			
Ethylbenzene	C_8H_{10}	100-41-4	Hydrocarbon/Raw material/Solvents/Fuel			√			
1-Octene, 6-methyl-	C_9H_{18}	13151-10-5	Hydrocarbon					√	
Octane, 4-methyl-	C_9H_{20}	2216-34-4	Hydrocarbon			√			
Hexanal, 5-methyl-	$C_7H_{14}O$	1860-39-5	Plastic related compounds		√			√	√
1-Hexanol	$C_6H_{14}O$	111-27-3	Fragrance/Plasticizers		√			√	
1-Butanol, 3-methyl-, acetate	$C_7H_{14}O_2$	123-92-2	Solvents			√			
Hexanal, 4-methyl-	$C_7H_{14}O$	41065-97-8	Plastic related compounds					√	
3-Hexanone, 5-methyl-	$C_7H_{14}O$	623-56-3	Plastic related compounds					√	
Nonane	C_9H_{20}	111-84-2	Hydrocarbon						√
Hexanal, 3-methyl-	$C_7H_{14}O$	19269-28-4	Fragrance			√			

2-Propenoic acid, anhydride	$C_6H_6O_3$	2051-76-5	Plastic related compounds						√
3-Heptanol, 4-methyl-	$C_8H_{18}O$	14979-39-6	Plastic related compounds					√	
meso-3,4-Hexanediol	$C_6H_{14}O_2$	22520-39-4	Plastic related compounds				√		
2,5-Hexanedione	$C_6H_{10}O_2$	110-13-4	Solvents						√
2-Octene, 2,6-dimethyl-	$C_{10}H_{20}$	4057-42-5	Hydrocarbon						√
2(5H)-Furanone, 5,5-dimethyl-	$C_6H_8O_2$	20019-64-1	Fragrance		√			√	√
2-Heptanone, 6-methyl-	$C_8H_{16}O$	928-68-7	Raw material/Fragrance					√	
1-Heptanol	$C_7H_{16}O$	111-70-6	Raw materials		√				
1-Hexanol, 2,2-dimethyl-	$C_8H_{18}O$	2370-13-0	"Hydrocarbon/Fragrance						√
1-Decene	$C_{10}H_{20}$	872-05-9	Plasticizers/Monomers"		√			√	
Decane	$C_{10}H_{22}$	124-18-5	Plastic related compounds						√
Octanal	$C_8H_{16}O$	124-13-0	Hydrocarbon/Fuel	√		√		√	
Benzene, 1-methyl-3-(1-methylethyl)-	$C_{10}H_{14}$	535-77-3	Fragrance		√				√
1-Hexanol, 2-ethyl-	$C_8H_{18}O$	104-76-7	Hydrocarbon/Solvents	√	√			√	
2(3H)-Furanone, 5-ethenyldihydro-5-methyl-	$C_7H_{10}O_2$	1073-11-6	Plasticizers		√			√	
2-Octen-1-ol	$C_8H_{16}O$	22104-78-5	Fragrance					√	
Nonane, 2,8-dimethyl-4-methylene-	$C_{12}H_{24}$	7323-15-1	Fragrance						
1-Octanol	$C_8H_{18}O$	111-87-5	Hydrocarbon					√	
Formic acid, 2-ethylhexyl ester	$C_9H_{18}O_2$	5460-45-7	Solvents						√
1-Undecene	$C_{11}H_{22}$	821-95-4	Plastic related compounds		√				
Undecane	$C_{11}H_{24}$	1120-21-4	Hydrocarbon				√		√
Nonanal	$C_9H_{18}O$	124-19-6	Hydrocarbon/Solvents	√	√				
2-Undecene, 9-methyl-, (Z)-	$C_{12}H_{24}$	74630-45-8	Fragrance					√	√
2-Nonen-1-ol, (E)-	$C_9H_{18}O$	31502-14-4	Hydrocarbon				√		
Levomenthol	$C_{10}H_{20}O$	2216-51-5	Fragrance		√			√	
Undecane, 3,5-dimethyl-	$C_{13}H_{28}$	17312-81-1	Fragrance					√	
Dodecane	$C_{12}H_{26}$	112-40-3	Hydrocarbon	√					
Decanal	$C_{10}H_{20}O$	112-31-2	Hydrocarbon/Solvents					√	√
3-Isopropylbenzaldehyde	$C_{10}H_{12}O$	34246-57-6	Fragrance		√				
Tetrahydrogeranyl formate	$C_{11}H_{22}O_2$	68214-06-2	Plastic related compounds					√	
2-Decenal, (E)-	$C_{10}H_{18}O$	3913-81-3	Plastic related compounds		√				
Undecane, 2,3-dimethyl-	$C_{13}H_{28}$	17312-77-5	Fragrance					√	
1-Tridecene	$C_{13}H_{26}$	2437-56-1	Hydrocarbon		√			√	
Tridecane	$C_{13}H_{28}$	629-50-5	Hydrocarbon/Intermediates		√				
Undecanal	$C_{11}H_{22}O$	112-44-7	Hydrocarbon/Raw material						√
Dodecane, 2,7,10-trimethyl-	$C_{15}H_{32}$	74645-98-0	Fragrance				√		
2-Methyl-1-undecanol	$C_{12}H_{26}O$	10522-26-6	Hydrocarbon						√

1-Tetradecene	$C_{14}H_{28}$	1120-36-1	Fragrance		√
Dodecanal	$C_{12}H_{24}O$	112-54-9	Hydrocarbon/Solvents		√
Pentadecane, 7-methyl-	$C_{16}H_{34}$	6165-40-8	Fragrance	√	
Hexadecane	$C_{16}H_{34}$	544-76-3	Hydrocarbon		√
Heptadecane	$C_{17}H_{36}$	629-78-7	Hydrocarbon/Solvents/Intermediate/Fragrance		√

Table S11. Potential sources of VOCs released from the 2# bottle

Compound	Formula	CAS	Source	Detected reaction					
				Dark-1 d	UV-A-1 d	Sunlight-1 d	Dark-7 d	UV-A-7 d	Sunlight-7 d
Acetone	C_3H_6O	67-64-1	Solvents /Intermediates	√	√	√		√	√
Formic acid	CH_2O_2	64-18-6	Plasticizers						√
1-Pentene, 2-methyl-	C_6H_{12}	763-29-1	Hydrocarbon			√		√	
n-Hexane	C_6H_{14}	110-54-3	Hydrocarbon/Additive/Solvents	√					
Ethyl Acetate	$C_4H_8O_2$	141-78-6	Solvents	√					
Acetic acid	$C_2H_4O_2$	64-19-7	Raw material		√	√			√
1-Heptene	C_7H_{14}	592-76-7	Hydrocarbon		√			√	
Pentanal	$C_5H_{10}O$	110-62-3	Fragrance		√				
Butanal, 2,2-dimethyl-	$C_6H_{12}O$	2094-75-9	Plastic related compounds				√		
n-Propyl acetate	$C_5H_{10}O_2$	109-60-4	Fragrance		√	√	√	√	√
Furan, 2-methoxy-	$C_5H_6O_2$	25414-22-6	Plastic related compounds				√		
Methyl Isobutyl Ketone	$C_6H_{12}O$	108-10-1	Adhesive/Solvents /Raw material				√		
sec-Butyl acetate	$C_6H_{12}O_2$	105-46-4	Solvents				√	√	
2-Pentanone, 4,4-dimethyl-	$C_7H_{14}O$	590-50-1	Solvents					√	√
1-Octene	C_8H_{16}	111-66-0	Hydrocarbon/Plasticizers		√		√		
Acetic acid, butyl ester	$C_6H_{12}O_2$	123-86-4	Solvents				√	√	
2-Hexene, 2,3-dimethyl-	C_8H_{16}	7145-20-2	Hydrocarbon			√			
1-Methylpentyl cyclopropane	C_9H_{18}	6976-28-9	Hydrocarbon						√
1-Octene, 6-methyl-	C_9H_{18}	13151-10-5	Hydrocarbon			√		√	√
4-Isopropoxy-2-butanone	$C_7H_{14}O_2$	32541-58-5	Plastic related compounds						√
1-Methoxy-2-propyl acetate	$C_6H_{12}O_3$	108-65-6	Solvents		√				
Hexanal, 4-methyl-	$C_7H_{14}O$	41065-97-8	Plastic related compounds						
3-Heptanone	$C_7H_{14}O$	106-35-4	Fragrance					√	√
2-Heptanone	$C_7H_{14}O$	110-43-0	Fragrance					√	
1-Nonene	C_9H_{18}	124-11-8	Hydrocarbon/Solvents		√			√	
1-Octene, 2,6-dimethyl-	$C_{10}H_{20}$	6874-29-9	Hydrocarbon						√
3-Heptanol, 4-methyl-	$C_8H_{18}O$	14979-39-6	Solvents					√	√

Propanoic acid, anhydride	$C_6H_{10}O_3$	123-62-6	dehydrating agent			√	√	
3-Hexanol, 4-ethyl-	$C_8H_{18}O$	19780-44-0	Plastic related compounds		√			
4-Nonene	C_9H_{18}	2198-23-4	Hydrocarbon	√			√	
Heptanal	$C_7H_{14}O$	111-71-7	Fragrance					√
1-Hexanol, 5-methyl-	$C_7H_{16}O$	627-98-5	Solvents				√	√
2(5H)-Furanone, 5,5-dimethyl-	$C_6H_8O_2$	20019-64-1	Plastic related compounds	√			√	
2-Heptanone, 6-methyl-	$C_8H_{16}O$	928-68-7	Fragrance		√			
1-Heptanol	$C_7H_{16}O$	111-70-6	Raw material/Fragrance	√				√
3-Pentenoic acid, 4-methyl-	$C_6H_{10}O_2$	504-85-8	Fragrance				√	√
1-Hepten-6-one, 2-methyl-	$C_8H_{14}O$	10408-15-8	Plastic related compounds			√		
1-Decene	$C_{10}H_{20}$	872-05-9	"Hydrocarbon/Fragrance		√			√
2-Hepten-4-one, 2-methyl-	$C_8H_{14}O$	22319-24-0	Plasticizers/Monomers"	√				
Octane, 3,4,5,6-tetramethyl-	$C_{12}H_{26}$	62185-21-1	Plastic related compounds					√
Octanal	$C_8H_{16}O$	124-13-0	Hydrocarbon	√				√
Ether, 6-methylheptyl vinyl	$C_{10}H_{20}O$	10573-35-0	Fragrance				√	
1-Hexanol, 2-ethyl-	$C_8H_{18}O$	104-76-7	Plastic related compounds	√	√			
2-Octen-1-ol	$C_8H_{16}O$	22104-78-5	Plasticizers		√			
4-Decene, 3-methyl-, (E)-	$C_{11}H_{22}$	62338-47-0	Fragrance	√				√
Acetophenone	C_8H_8O	98-86-2	Hydrocarbon	√	√			
1-Octanol	$C_8H_{18}O$	111-87-5	Solvents		√		√	
Formic acid, 2-ethylhexyl ester	$C_9H_{18}O_2$	5460-45-7	Solvents	√			√	√
1-Undecene	$C_{11}H_{22}$	821-95-4	Plastic related compounds	√			√	
Undecane	$C_{11}H_{24}$	1120-21-4	Hydrocarbon	√	√		√	
Nonanal	$C_9H_{18}O$	124-19-6	Hydrocarbon/Solvents	√	√			
(S)-(+)-6-Methyl-1-octanol	$C_9H_{20}O$	110453-78-6	Fragrance					√
Acetic acid, 2-ethylhexyl ester	$C_{10}H_{20}O_2$	103-09-3	Plastic related compounds	√			√	
1-Octanol, 2,7-dimethyl-	$C_{10}H_{22}O$	15250-22-3	Fragrance/Solvents					√
2-Nonenal, (E)-	$C_9H_{16}O$	18829-56-6	Plastic related compounds		√			
1-Undecene, 9-methyl-	$C_{12}H_{24}$	74630-41-4	Fragrance					√
1-Nonanol	$C_9H_{20}O$	143-08-8	Hydrocarbon		√		√	√
Levomenthol	$C_{10}H_{20}O$	2216-51-5	Fragrance/Plasticizers					√

Dodecane	$C_{12}H_{26}$	112-40-3	Fragrance	√	√			√
Decane, 6-ethyl-2-methyl-	$C_{13}H_{28}$	62108-21-8	Hydrocarbon/Solvents			√		
Decanal	$C_{10}H_{20}O$	112-31-2	Hydrocarbon		√		√	
Tetrahydrogeranyl formate	$C_{11}H_{22}O_2$	68214-06-2	Fragrance		√		√	
2-Decenal, (E)-	$C_{10}H_{18}O$	3913-81-3	Plastic related compounds	√				
1-Decanol	$C_{10}H_{22}O$	112-30-1	Fragrance		√			
1-Tridecene	$C_{13}H_{26}$	2437-56-1	Fragrance/Plasticizers		√			√
Dodecane, 2,6,11-trimethyl-	$C_{15}H_{32}$	31295-56-4	Hydrocarbon/Intermediates				√	
Tridecane	$C_{13}H_{28}$	629-50-5	Hydrocarbon	√	√			
2-Undecenal	$C_{11}H_{20}O$	2463-77-6	Hydrocarbon/Raw material		√			
1-Undecanol	$C_{11}H_{24}O$	112-42-5	Fragrance	√	√			√
1-Tetradecene	$C_{14}H_{28}$	1120-36-1	Fragrance				√	
Tetradecane	$C_{14}H_{30}$	629-59-4	Hydrocarbon/Solvents	√	√			√
Dodecanal	$C_{12}H_{24}O$	112-54-9	Hydrocarbon/Solvents		√		√	√
1,9-Nonanediol	$C_9H_{20}O_2$	3937-56-2	Fragrance	√				
1-Dodecanol	$C_{12}H_{26}O$	112-53-8	Plastic related compounds				√	√
Pentadecane	$C_{15}H_{32}$	629-62-9	Fragrance/Plasticizers		√		√	
Hexadecane	$C_{16}H_{34}$	544-76-3	Hydrocarbon/Solvents		√		√	√
Heptadecane	$C_{17}H_{36}$	629-78-7	Hydrocarbon/Solvents/Intermediate/Fragrance	√	√			

Table S12. Potential sources of VOCs released from the 3# bottle

Compound	Formula	CAS	Source	Detected reaction					
				Dark-1 d	UV-A-1 d	Sunlight-1 d	Dark-7 d	UV-A-7 d	Sunlight-7 d
2-Butanol, 2,3-dimethyl-	$C_6H_{14}O$	594-60-5	Plastic related compounds					√	
1-Pentanol	$C_5H_{12}O$	71-41-0	Plasticizers/Fragrance/Solvents					√	
Butane, 2,2,3,3-tetramethyl-	C_8H_{18}	594-82-1	Hydrocarbon					√	
Pentane, 2,2,3-trimethyl-	C_8H_{18}	564-02-3	Hydrocarbon		√				
Propanoic acid, 2-methyl-	$C_4H_8O_2$	79-31-2	Fragrance	√		√	√		√
Pentanal, 2-methyl-	$C_6H_{12}O$	123-15-9	Fragrance/Intermediates					√	
Toluene	C_7H_8	108-88-3	Hydrocarbon/Adhesive					√	√
Pentane, 2,2,4,4-tetramethyl-	C_9H_{20}	1070-87-7	Plastic related compounds						√
1-Octene	C_8H_{16}	111-66-0	Hydrocarbon/Plasticizers					√	
1-Heptene, 3-methyl-	C_8H_{16}	4810-09-7	Hydrocarbon			√			
Octane	C_8H_{18}	111-65-9	Hydrocarbon/Solvents			√		√	√
2-Pentanone, 3-ethyl-	$C_7H_{14}O$	6137-03-7	Plastic related compounds					√	
2-Hexanone, 3-methyl-	$C_7H_{14}O$	2550-21-2	Plastic related compounds			√			
Butanoic acid, 3-methyl-	$C_5H_{10}O_2$	503-74-2	Plasticizers/Fragrance	√			√		√
Butanoic acid, 2-methyl-	$C_5H_{10}O_2$	116-53-0	Fragrance	√					√
Heptane, 2,5,5-trimethyl-	$C_{10}H_{22}$	1189-99-7	Hydrocarbon			√			
Formic acid, hexyl ester	$C_7H_{14}O_2$	629-33-4	Fragrance					√	
Hexanal, 5-methyl-	$C_7H_{14}O$	1860-39-5	Plastic related compounds						√
Benzene, 1,3-dimethyl-	C_8H_{10}	108-38-3	Hydrocarbon/Solvents	√			√		
Hexanal, 4-methyl-	$C_7H_{14}O$	41065-97-8	Plastic related compounds						√
3-Heptanone	$C_7H_{14}O$	106-35-4	Fragrance						√
Pentanoic acid	$C_5H_{10}O_2$	109-52-4	Plasticizers/Fragrance						√
1-Nonene	C_9H_{18}	124-11-8	Hydrocarbon/Solvents			√			√
Cyclohexanone	$C_6H_{10}O$	108-94-1	Hydrocarbon					√	
Nonane	C_9H_{20}	111-84-2	Hydrocarbon		√				
Hexane, 2,2,3,3-tetramethyl-	$C_{10}H_{22}$	13475-81-5	Fragrance						√
Hexanal, 3-methyl-	$C_7H_{14}O$	19269-28-4	Fragrance	√					
Heptanal	$C_7H_{14}O$	111-71-7	Plastic related compounds						√

meso-3,4-Hexanediol	$C_6H_{14}O_2$	22520-39-4	Hydrocarbon				√		
4-Nonene	C_9H_{18}	2198-23-4	Plastic related compounds						√
1-Hydroxy-2-pentanone	$C_5H_{10}O_2$	64502-89-2	Fragrance		√				
Ethanone, 1-cyclopentyl-	$C_7H_{12}O$	6004-60-0	Fragrance			√			
2(5H)-Furanone, 5,5-dimethyl-	$C_6H_8O_2$	20019-64-1	Plastic related compounds						√
2-Heptanone, 6-methyl-	$C_8H_{16}O$	928-68-7	Plastic related compounds						√
Hexanal, 2-ethyl-	$C_8H_{16}O$	123-05-7	Plastic related compounds			√		√	
4-Oxohex-2-enal	$C_6H_8O_2$	20697-55-6	Plastic related compounds						√
Heptane, 3,3,4-trimethyl-	$C_{10}H_{22}$	20278-87-9	Hydrocarbon		√				
1-Heptanol	$C_7H_{16}O$	111-70-6	Raw material/Fragrance			√	√		√
Hexanoic acid	$C_6H_{12}O_2$	142-62-1	Fragrance				√		
5-Hepten-2-one, 6-methyl-	$C_8H_{14}O$	110-93-0	Fragrance	√			√		
Hexanal, 2,2-dimethyl-	$C_8H_{16}O$	996-12-3	Plastic related compounds					√	
4-Penten-1-ol, propanoate	$C_8H_{14}O_2$	30563-30-5	Plastic related compounds					√	
1-Decene	$C_{10}H_{20}$	872-05-9	"Hydrocarbon/Fragrance						√
Decane	$C_{10}H_{22}$	124-18-5	Plasticizers/Monomers"					√	
3,4-Hexanedione, 2,2,5-trimethyl-	$C_9H_{16}O_2$	20633-03-8	Hydrocarbon/Fuel		√	√			
Nonane, 4-methyl-	$C_{10}H_{22}$	17301-94-9	Plastic related compounds						√
Octanal	$C_8H_{16}O$	124-13-0	Hydrocarbon			√			√
1-Decene, 8-methyl-	$C_{11}H_{22}$	61142-79-8	Fragrance					√	
Pentane, 1-(1-butenyloxy)-, (E)-	$C_9H_{18}O$	54004-25-0	Hydrocarbon						√
1-Hexanol, 2-ethyl-	$C_8H_{18}O$	104-76-7	Plastic related compounds	√	√		√		
1-Propanol, 2-(2-hydroxypropoxy)-	$C_6H_{14}O_3$	106-62-7	Plasticizers	√		√	√		√
2,7-Octanedione	$C_8H_{14}O_2$	1626-09-1	Adhesive/Plasticizers/Solvents						√
4-Decene, 3-methyl-, (E)-	$C_{11}H_{22}$	62338-47-0	Plastic related compounds						√
Acetophenone	C_8H_8O	98-86-2	Hydrocarbon				√	√	√
1-Octanol	$C_8H_{18}O$	111-87-5	Solvents	√	√				√
3-Nonanone	$C_9H_{18}O$	925-78-0	Solvents						√
4-Decene, 4-methyl-, (E)-	$C_{11}H_{22}$	60366-66-7	Fragrance						√
6-Hepten-3-one, 5-hydroxy-4,6-dimethyl-	$C_9H_{16}O_2$	62338-59-4	Hydrocarbon						√
Nonanal	$C_9H_{18}O$	124-19-6	Plastic related compounds		√	√		√	√

2-Nonen-1-ol	$C_9H_{18}O$	22104-79-6	Fragrance				√		
Hexanoic acid, 2-ethyl-	$C_8H_{16}O_2$	149-57-5	Fragrance						√
Formic acid, octyl ester	$C_9H_{18}O_2$	112-32-3	Plasticizers						√
Undecane, 5-methylene-	$C_{12}H_{24}$	5698-48-6	Fragrance						√
l-Menthone	$C_{10}H_{18}O$	14073-97-3	Hydrocarbon	√					
1-Nonanol	$C_9H_{20}O$	143-08-8	Fragrance			√		√	
Levomenthol	$C_{10}H_{20}O$	2216-51-5	Fragrance/Plasticizers	√					
Dodecane	$C_{12}H_{26}$	112-40-3	Fragrance						√
Butanoic acid, 4-hexenyl ester, (Z)-	$C_{10}H_{18}O_2$	69727-41-9	Hydrocarbon/Solvents					√	
Decanal	$C_{10}H_{20}O$	112-31-2	Plastic related compounds	√	√		√		√
1-(2,3-Dimethylphenyl)ethanone	$C_{10}H_{12}O$	2142-71-4	Fragrance			√			
2-Ethylhexyl acrylate	$C_{11}H_{20}O_2$	103-11-7	Plastic related compounds						√
1-Decanol	$C_{10}H_{22}O$	112-30-1	Plasticizers/Monomers						√
Nonane, 5-methyl-5-propyl-	$C_{13}H_{28}$	62238-11-3	Fragrance/Plasticizers						√
Nonanoic acid	$C_9H_{18}O_2$	112-05-0	Hydrocarbon						√
Tridecane	$C_{13}H_{28}$	629-50-5	Fragrance/Adhesive			√			√
1-Undecanol	$C_{11}H_{24}O$	112-42-5	Hydrocarbon/Raw material						√
Tetradecane	$C_{14}H_{30}$	629-59-4	Fragrance	√	√	√	√		
Dodecanal	$C_{12}H_{24}O$	112-54-9	Hydrocarbon/Solvents						√
Pentadecane	$C_{15}H_{32}$	629-62-9	Fragrance					√	√
Hexadecane	$C_{16}H_{34}$	544-76-3	Hydrocarbon/Solvents	√		√		√	√
Heptadecane	$C_{17}H_{36}$	629-78-7	Hydrocarbon/Solvents/Intermediate/Fragrance						√

Table S13. Potential sources of VOCs released from the 4# bottle

Compound	Formula	CAS	Source	Detected reaction					
				Dark-1 d	UV-A-1 d	Sunlight-1 d	Dark-7 d	UV-A-7 d	Sunlight-7 d
Isopropyl Alcohol	C_3H_8O	67-63-0	Solvents	√					
Acetic acid	$C_2H_4O_2$	64-19-7	Raw material						√
Propanal, 2-methyl-	C_4H_8O	78-84-2	Fragrance/Plasticizers			√			
1-Heptene	C_7H_{14}	592-76-7	Hydrocarbon			√		√	√
Acetylacetone	$C_5H_8O_2$	123-54-6	Solvents/Fuel					√	
Methyl Isobutyl Ketone	$C_6H_{12}O$	108-10-1	Adhesive/Solvents /Raw material			√			√
Propanoic acid, 2-methyl-	$C_4H_8O_2$	79-31-2	Fragrance					√	
1-Heptene, 6-methyl-	C_8H_{16}	5026-76-6	Hydrocarbon						√
1-Hexene, 3,3,5-trimethyl-	C_9H_{18}	13427-43-5	Hydrocarbon		√	√			
Toluene	C_7H_8	108-88-3	Hydrocarbon					√	
Pentane, 2,2,3-trimethyl-	C_8H_{18}	564-02-3	Hydrocarbon			√			
2-Pentanone, 4,4-dimethyl-	$C_7H_{14}O$	590-50-1	Solvents					√	
1-Octene	C_8H_{16}	111-66-0	Hydrocarbon/Plasticizers		√				√
Octane	C_8H_{18}	111-65-9	Hydrocarbon/Solvents			√			√
1-Pentanol, 3-methyl-	$C_6H_{14}O$	589-35-5	Fragrance		√			√	
1-Hepten-3-one	$C_7H_{12}O$	2918-13-0	Plastic related compounds					√	
2-Pentanone, 3-ethyl-3-methyl-	$C_8H_{16}O$	19780-65-5	Plastic related compounds						√
3-Heptanone	$C_7H_{14}O$	106-35-4	Fragrance					√	
1-Nonene	C_9H_{18}	124-11-8	Hydrocarbon/Solvents		√			√	√
1-Hexanol, 3-methyl-	$C_7H_{16}O$	13231-81-7	Plastic related compounds			√			
Heptanal	$C_7H_{14}O$	111-71-7	Fragrance					√	
2(5H)-Furanone, 5,5-dimethyl-	$C_6H_8O_2$	20019-64-1	Plastic related compounds					√	
2-Heptanone, 6-methyl-	$C_8H_{16}O$	928-68-7	Fragrance		√			√	
Benzaldehyde	C_7H_6O	100-52-7	Solvents/Fragrance					√	
1-Heptanol	$C_7H_{16}O$	111-70-6	Raw material/Fragrance						√
1-Heptanol, 6-methyl-	$C_8H_{18}O$	1653-40-3	Plasticizers						√

1-Decene	$C_{10}H_{20}$	872-05-9	"Hydrocarbon/Fragrance		√	√		
3,4-Hexanedione, 2,2,5-trimethyl-	$C_9H_{16}O_2$	20633-03-8	Plasticizers/Monomers"		√			
Decane	$C_{10}H_{22}$	124-18-5	Plastic related compounds				√	
Octanal	$C_8H_{16}O$	124-13-0	Hydrocarbon/Fuel					√
1-Hexanol, 2-ethyl-	$C_8H_{18}O$	104-76-7	Fragrance	√		√		
1-Octanol	$C_8H_{18}O$	111-87-5	Plasticizers			√		√
(S)-(+)-6-Methyl-1-octanol	$C_9H_{20}O$	110453-78-6	Solvents					√
Nonanal	$C_9H_{18}O$	124-19-6	Plastic related compounds			√		
1-Nonanol	$C_9H_{20}O$	143-08-8	Fragrance		√		√	√
5-Undecene, 6-methyl-	$C_{12}H_{24}$	83687-45-0	Fragrance/Plasticizers		√	√		√
Dodecane	$C_{12}H_{26}$	112-40-3	Hydrocarbon				√	
Decanal	$C_{10}H_{20}O$	112-31-2	Hydrocarbon/Solvents	√		√	√	
1-Tridecene	$C_{13}H_{26}$	2437-56-1	Fragrance			√		
Tridecane	$C_{13}H_{28}$	629-50-5	Hydrocarbon/Intermediates		√			
Undecanal	$C_{11}H_{22}O$	112-44-7	Hydrocarbon/Raw material				√	
Pentadecane	$C_{15}H_{32}$	629-62-9	Fragrance				√	

Table S14. Potential sources of VOCs released from the 5# bottle

Compound	Formula	CAS	Source	Detected reaction					
				Dark-1 d	UV-A-1 d	Sunlight-1 d	Dark-7 d	UV-A-7 d	Sunlight-7 d
Acetic acid	$C_2H_4O_2$	64-19-7	Raw material						√
1-Hexene	C_6H_{12}	592-41-6	Hydrocarbon/Monomers/Fragrance			√			
Propanal, 2-methyl-	C_4H_8O	78-84-2	Fragrance/Plasticizers		√				
3-Methyl-3-hexene	C_7H_{14}	3404-65-7	Hydrocarbon		√	√		√	√
3-Pentanone, 2-methyl-	$C_6H_{12}O$	565-69-5	Raw material						√
Acetylacetone	$C_5H_8O_2$	123-54-6	Solvents/Fuel						√
1-Penten-3-one, 2,4-dimethyl-	$C_7H_{12}O$	3212-68-8	Plastic related compounds		√				
1-Heptene, 6-methyl-	C_8H_{16}	5026-76-6	Hydrocarbon						√
1-Hexene, 3,3,5-trimethyl-	C_9H_{18}	13427-43-5	Hydrocarbon					√	
1-Octene	C_8H_{16}	111-66-0	Hydrocarbon/Plasticizers			√			√
Octane	C_8H_{18}	111-65-9	Hydrocarbon/Solvents		√				√
Butanal, 3,3-dimethyl-2-oxo-, hemihydrate	$C_6H_{10}O_2$	4480-47-1	Plastic related compounds		√				
2-Pentanone, 3-ethyl-3-methyl-	$C_8H_{16}O$	19780-65-5	Plastic related compounds						√
1-Hexanol	$C_6H_{14}O$	111-27-3	Fragrance/Plasticizers						√
3-Heptanone	$C_7H_{14}O$	106-35-4	Fragrance					√	√
1-Hexanol, 3-methyl-	$C_7H_{16}O$	13231-81-7	Plastic related compounds		√	√			
1-Nonene	C_9H_{18}	124-11-8	Hydrocarbon/Solvent			√			
3-Heptanol	$C_7H_{16}O$	589-82-2	Fragrance						√
Nonane	C_9H_{20}	111-84-2	Hydrocarbon		√				
Heptanal	$C_7H_{14}O$	111-71-7	Fragrance					√	√
2(5H)-Furanone, 5,5-dimethyl-	$C_6H_8O_2$	20019-64-1	Plastic related compounds					√	
Hexanal, 2-ethyl-	$C_8H_{16}O$	123-05-7	Fragrance			√			
4-Nonene, 3-methyl-, (Z)-	$C_{10}H_{20}$	63830-69-3	Hydrocarbon						√
1-Heptanol	$C_7H_{16}O$	111-70-6	Raw material/Fragrance			√		√	√
1-Heptanol, 6-methyl-	$C_8H_{18}O$	1653-40-3	Plasticizers					√	
3-Decene	$C_{10}H_{20}$	19398-37-9	Hydrocarbon		√				

Octanal	$C_8H_{16}O$	124-13-0	Fragrance			√	
3,6-Heptanedione	$C_7H_{12}O_2$	1703-51-1	Plastic related compounds				√
1-Hexanol, 2-ethyl-	$C_8H_{18}O$	104-76-7	Plasticizers				√
2-Decene, 9-methyl-, (Z)-	$C_{11}H_{22}$	74630-24-3	Hydrocarbon				√
Acetophenone	C_8H_8O	98-86-2	Solvents				√
1-Octanol	$C_8H_{18}O$	111-87-5	Solvents		√		√
Undecane	$C_{11}H_{24}$	1120-21-4	Hydrocarbon/Solvents			√	√
Formic acid, octyl ester	$C_9H_{18}O_2$	112-32-3	Fragrance				√
1-Nonanol	$C_9H_{20}O$	143-08-8	Fragrance/Plasticizers			√	
Undecane, 2,8-dimethyl-	$C_{13}H_{28}$	17301-25-6	Hydrocarbon	√			
Dodecane	$C_{12}H_{26}$	112-40-3	Hydrocarbon/Solvents				√
Decanal	$C_{10}H_{20}O$	112-31-2	Fragrance	√		√	√
1-Tridecene	$C_{13}H_{26}$	2437-56-1	Hydrocarbon/Intermediates				√

Table S15. Potential sources of VOCs released from the 6# bottle

Compound	Formula	CAS	Source	Detected reaction					
				Dark-1 d	UV-A-1 d	Sunlight-1 d	Dark-7 d	UV-A-7 d	Sunlight-7 d
Isopropyl Alcohol	C_3H_8O	67-63-0	Solvents		√		√		
Butane, 2-methoxy-	$C_5H_{12}O$	6795-87-5	Plastic related compounds		√				
1-Pentene, 2-methyl-	C_6H_{12}	763-29-1	Hydrocarbon					√	√
3-Methyl-3-hexene	C_7H_{14}	3404-65-7	Hydrocarbon					√	√
2-Butanone, 3,3-dimethyl-	$C_6H_{12}O$	75-97-8	Solvents		√				
Methyl Isobutyl Ketone	$C_6H_{12}O$	108-10-1	Adhesive/Solvents /Raw material		√				
Acetylacetone	$C_5H_8O_2$	123-54-6	Solvents/Fuel					√	
1-Hexene, 3,3,5-trimethyl-	C_9H_{18}	13427-43-5	Hydrocarbon		√				
1-Heptene, 6-methyl-	C_8H_{16}	5026-76-6	Hydrocarbon					√	
1-Octene	C_8H_{16}	111-66-0	Hydrocarbon/Plasticizers			√		√	
Hexanal	$C_6H_{12}O$	66-25-1	Fragrance	√	√				
2-Hexene, 2,3-dimethyl-	C_8H_{16}	7145-20-2	Hydrocarbon					√	
Heptane, 2,3-dimethyl-	C_9H_{20}	3074-71-3	Hydrocarbon					√	
Pentane, 2,3,3,4-tetramethyl-	C_9H_{20}	16747-38-9	Hydrocarbon		√				
3-Heptanone	$C_7H_{14}O$	106-35-4	Fragrance		√	√		√	
1-Nonene	C_9H_{18}	124-11-8	Hydrocarbon/Solvents		√				
Hexanal, 3-methyl-	$C_7H_{14}O$	19269-28-4	Fragrance					√	√
1-Hexanol, 4-methyl-	$C_7H_{16}O$	818-49-5	Plastic related compounds					√	
2-Heptenol	$C_7H_{14}O$	33467-76-4	Plastic related compounds					√	
2H-Pyran-2-carboxaldehyde, 5,6-dihydro-	$C_6H_8O_2$	53897-26-0	Plastic related compounds		√			√	

2(5H)-Furanone, 5,5-dimethyl-	$C_6H_8O_2$	20019-64-1	Plastic related compounds					√	
Hexanal, 2-ethyl-	$C_8H_{16}O$	123-05-7	Fragrance				√		√
Benzaldehyde	C_7H_6O	100-52-7	Solvents/Fragrance		√				
1-Heptanol	$C_7H_{16}O$	111-70-6	Raw material/Fragrance		√	√			√
3-Pentenoic acid, 4-methyl-	$C_6H_{10}O_2$	504-85-8	Fragrance					√	
5-Hepten-2-one, 6-methyl-	$C_8H_{14}O$	110-93-0	Fragrance	√					
2-Octanone	$C_8H_{16}O$	111-13-7	Fragrance					√	
1-Decene	$C_{10}H_{20}$	872-05-9	"Hydrocarbon/Fragrance			√		√	√
2-Hexenal, 2-ethyl-	$C_8H_{14}O$	645-62-5	Plasticizers/Monomers"			√			
Decane	$C_{10}H_{22}$	124-18-5	Intermediates					√	
Octanal	$C_8H_{16}O$	124-13-0	Hydrocarbon/Fuel				√	√	
3,6-Heptanedione	$C_7H_{12}O_2$	1703-51-1	Fragrance					√	
1-Hexanol, 2-ethyl-	$C_8H_{18}O$	104-76-7	Plastic related compounds		√	√		√	
2-Decene, 6-methyl-, (Z)-	$C_{11}H_{22}$	74630-31-2	Plasticizers					√	
4-Decene, 3-methyl-, (E)-	$C_{11}H_{22}$	62338-47-0	Hydrocarbon					√	
Acetophenone	C_8H_8O	98-86-2	Hydrocarbon		√			√	
1-Octanol	$C_8H_{18}O$	111-87-5	Solvents			√			
Formic acid, 2-ethylhexyl ester	$C_9H_{18}O_2$	5460-45-7	Solvents		√	√		√	
3-Nonanone	$C_9H_{18}O$	925-78-0	Plastic related compounds					√	
1-Octanol, 2-methyl-	$C_9H_{20}O$	818-81-5	Fragrance					√	
1-Undecene	$C_{11}H_{22}$	821-95-4	Fragrance		√	√			
Undecane	$C_{11}H_{24}$	1120-21-4	Hydrocarbon			√		√	
Nonanal	$C_9H_{18}O$	124-19-6	Hydrocarbon/Solvents					√	

Hexanoic acid, 2-ethyl-	$C_8H_{16}O_2$	149-57-5	Fragrance	√		
Formic acid, octyl ester	$C_9H_{18}O_2$	112-32-3	Plasticizers			√
Acetic acid, 2-ethylhexyl ester	$C_{10}H_{20}O_2$	103-09-3	Fragrance			√
1-Nonanol	$C_9H_{20}O$	143-08-8	Fragrance/Solvents			√
5-Undecene, 6-methyl-	$C_{12}H_{24}$	83687-45-0	Fragrance/Plasticizers	√	√	
Dodecane	$C_{12}H_{26}$	112-40-3	Hydrocarbon			√
Decanal	$C_{10}H_{20}O$	112-31-2	Hydrocarbon/Solvents		√	√
Nonanoic acid	$C_9H_{18}O_2$	112-05-0	Fragrance/Adhesive			√
2-Dodecene, 2-methyl-	$C_{13}H_{26}$	55103-82-7	Plasticizers			√
Tridecane	$C_{13}H_{28}$	629-50-5	Hydrocarbon	√		√
Tetradecane	$C_{14}H_{30}$	629-59-4	Hydrocarbon/Raw material			√

Table S16. Top five VOCs based on TOXPi analysis

Ranking	1#	2#	3#	4#	5#	6#
1	Benzene	Hexadecane	Hexadecane	Pentadecane	Dodecane	Tridecane
2	Heptadecane	Heptadecane	Heptadecane	Tridecane	Decanal	Tetradecane
3	Hexadecane	Pentadecane	Pentadecane	Undecanal	1-Tridecene	Dodecane
4	Dodecanal	1-Dodecanol	Dodecanal	Dodecane	Formic acid, octyl ester	Decanal
5	1-Tetradecene	1-Tetradecene	Tridecane	Decanal	Undecane, 2,8-dimethyl-	2-Dodecene, 2-methyl-

Table S17. Yield of top five toxic VOCs from empty bottles.

Bottle number	Compounds	Dark-1 d	UV-A-1 d	Sunlight-1 d	Dark-7 d	UV-A-7 d	Sunlight-7 d
1#	Benzene	0	0	0.62	0	0	0
	Hexadecane	0	0	0	0	0.18	0
	Heptadecane	0	0	0	0	0.11	0
	Dodecanal	0	0	0	0	0.23	0
	Ethylbenzene	0	0	0	0	0.24	0
2#	Hexadecane	0	0	0.22	0	0.21	0.75
	Heptadecane	0	0.13	0.19	0	0	0
	Pentadecane	0	0	0.36	0	0.17	0
	1-Dodecanol	0	0	0	0	0.44	0.39
	1-Tetradecene	0	0	0	0	0.11	0
3#	Hexadecane	0.03	0	0.17	0	0.18	0.59
	Heptadecane	0	0	0	0	0	0.68
	Pentadecane	0	0	0	0	0.11	0.47
	Dodecanal	0	0	0	0	0	0.79
	Tridecane	0	0	0.17	0	0	0.04
4#	Pentadecane	0	0	0	0	0.08	0
	Tridecane	0	0.04	0	0	0	0
	Undecanal	0	0	0	0	0.03	0
	Dodecane	0	0	0	0	0.05	0
	Decanal	0.02	0	0.06	0	0.02	0
5#	Dodecane	0	0	0	0	0	0.06
	Decanal	0	0.09	0	0	0.18	0.16
	1-Tridecene	0	0	0	0	0	0.04
	Formic acid, octyl ester	0	0	0	0	0	0.19
	Undecane, 2,8-dimethyl-	0	0.07	0	0	0	0
6#	Tridecane	0	0.07	0	0	0.32	0

	Tetradecane	0	0	0	0	0.11	0
	Dodecane	0	0	0	0	0.45	0
	Decanal	0	0	0.08	0	0.16	0
	2-Dodecene, 2-methyl-	0	0	0	0	0.09	0

Table S18. Yield of top five toxic VOCs from 2# and 6# bottles with solution matrices.

Bottle number	Compounds	Dark-7 d			UV-A-7 d			Sunlight-7 d		
		Deionized water	Mineral water	Soda water	Deionized water	Mineral water	Soda water	Deionized water	Mineral water	Soda water
	Hexadecane	0	0	0	0.19	0.28	0	0.75	0.89	0.31
	Heptadecane	0	0	0	0	0	0	0	0	0
2#	Pentadecane	0	0	0	0.21	0.31	0	0	0	0
	1-Dodecanol	0	0	0	0.46	0.65	0.16	0.39	0.42	0.11
	1-Tetradecene	0	0	0	0.17	0.28	0	0	0	0
	Tridecane	0	0	0	0.24	0.59	0.16	0	0	0
	Tetradecane	0	0	0	0.13	0.31	0	0	0	0
6#	Dodecane	0	0	0	0.49	0.72	0.15	0	0	0
	Decanal	0	0	0	0.17	0.34	0.08	0	0	0
	2-Dodecene, 2-methyl-	0	0	0	0.08	0.16	0	0	0	0

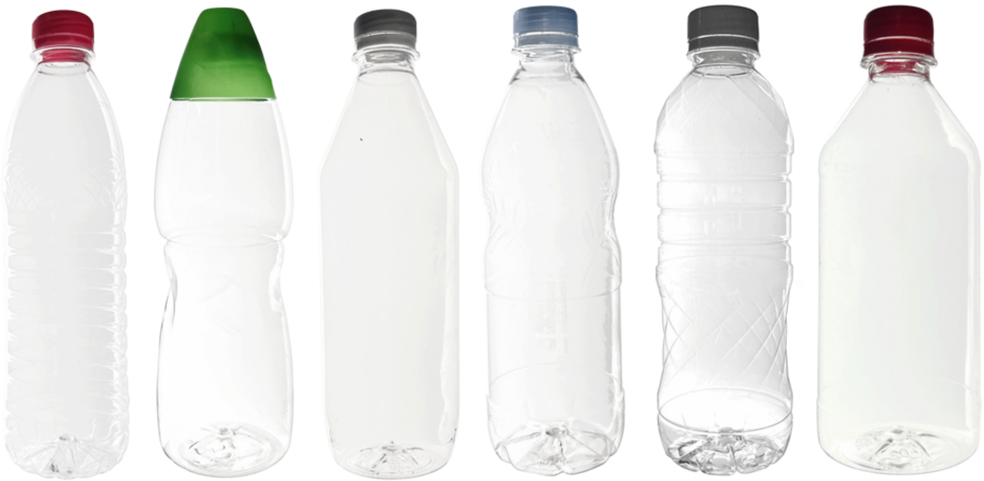

Figure S1. Images of bottles

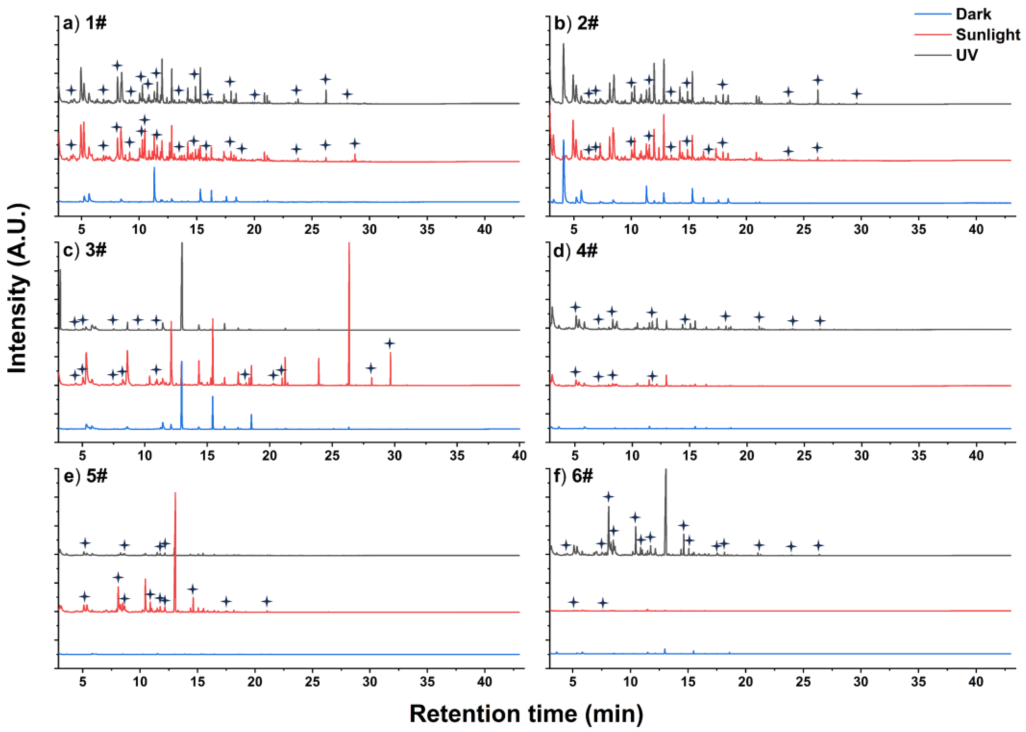

Figure S2. Total ion chromatogram of VOCs from bottles after long-term treatments. a)-f) were #1-#6 bottles.

UV-A irradiation intensity: 5.6 W m^{-2}, reaction time 12 h per day; Sunlight irradiation dose: 5.6 W m^{-2}, irradiation time 12 h per day. Short-term experiment is conducted for 1 d, while long-term experiment is conducted for 7 d. Asterisk Mark indicates new peaks of the treated samples, which were not observed in the dark samples but could be observed in the treated samples. Only some peaks are labeled.
